# Supplementary figures and images for: Aerobactin is a key driver of hypervirulent Klebsiella pneumoniae translocation and virulence
Source: PLoS Pathog. 2026 Apr 13;22(4):e1014122. doi: 10.1371/journal.ppat.1014122 (PMC13089870; doi:10.1371/journal.ppat.1014122)

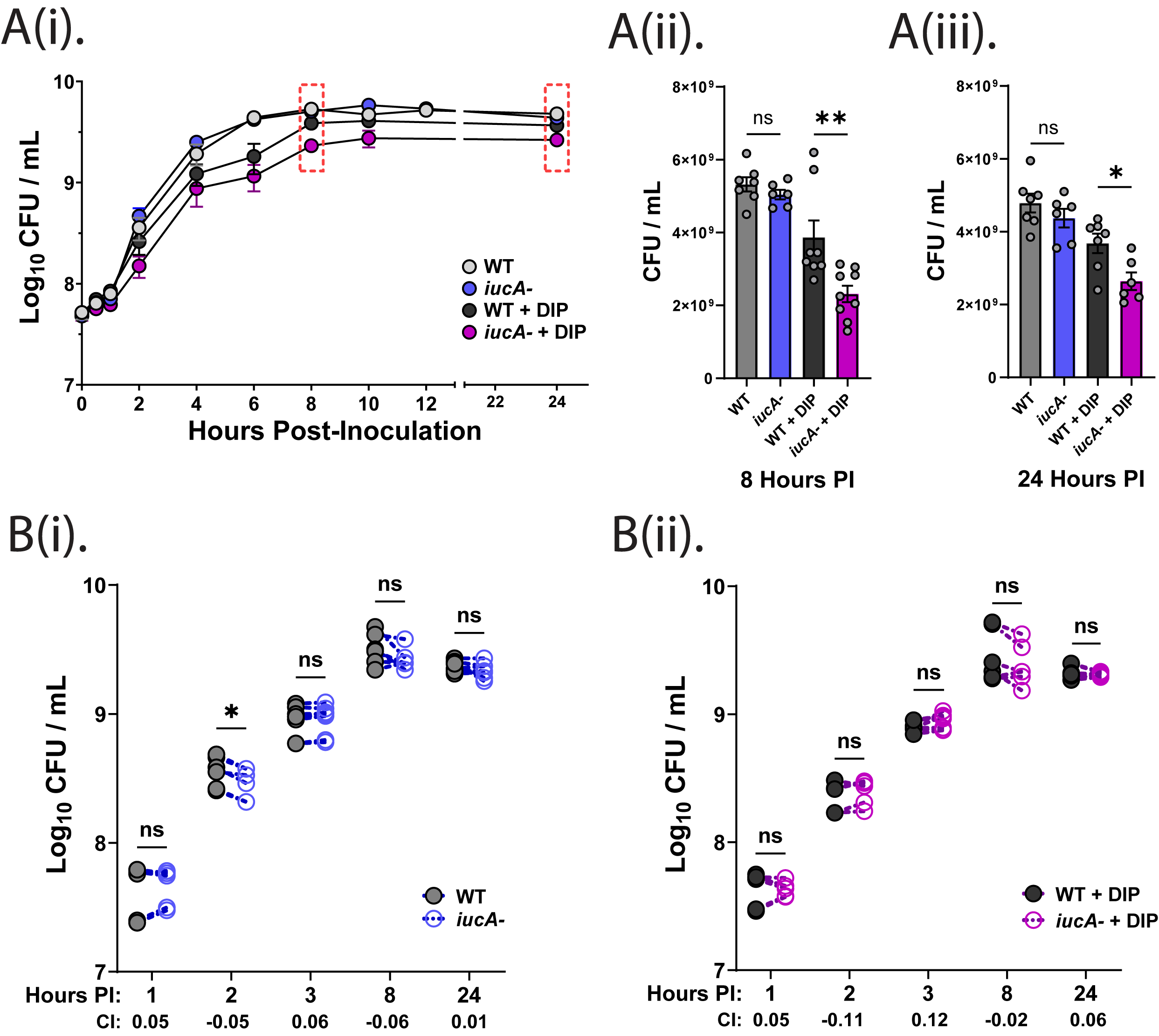

Supplement: S1 Fig — (A) Growth kinetics of the WT and iucA- mutant grown in LB (iron-replete media) or LB + 200 µM DIP (iron-chelated media) at 37°C under aerobic conditions, shown as (i) log10 CFU/mL over time. CFU counts for the WT and iucA- mutant in each growth condition were compared at 8 hours (i) and 24 hours (ii) PI using Mann-Whitney U tests. (B) An in vitro competition assay between the WT (gray circles) and the iucA- (blue open circles) inoculated 1:1 in (i) LB or (ii) LB + 200 µM DIP. CFU were enumerated for the WT and the iucA- mutant for each biological replicate (n ≥ 6) at 1, 2, 3, 8, and 24 hours PI. Dotted lines connect WT and mutant CFU counts recovered from the same biological replicate. The Wilcoxon matched-pairs signed rank test was employed to determine statistical significance. Competitive Index (CI) values were calculated for each time point as described in the Materials and Methods. PI, post-inoculation. *P < 0.05, **P < 0.01; ns, not significant. (TIF) [file ppat.1014122.s001.tif]

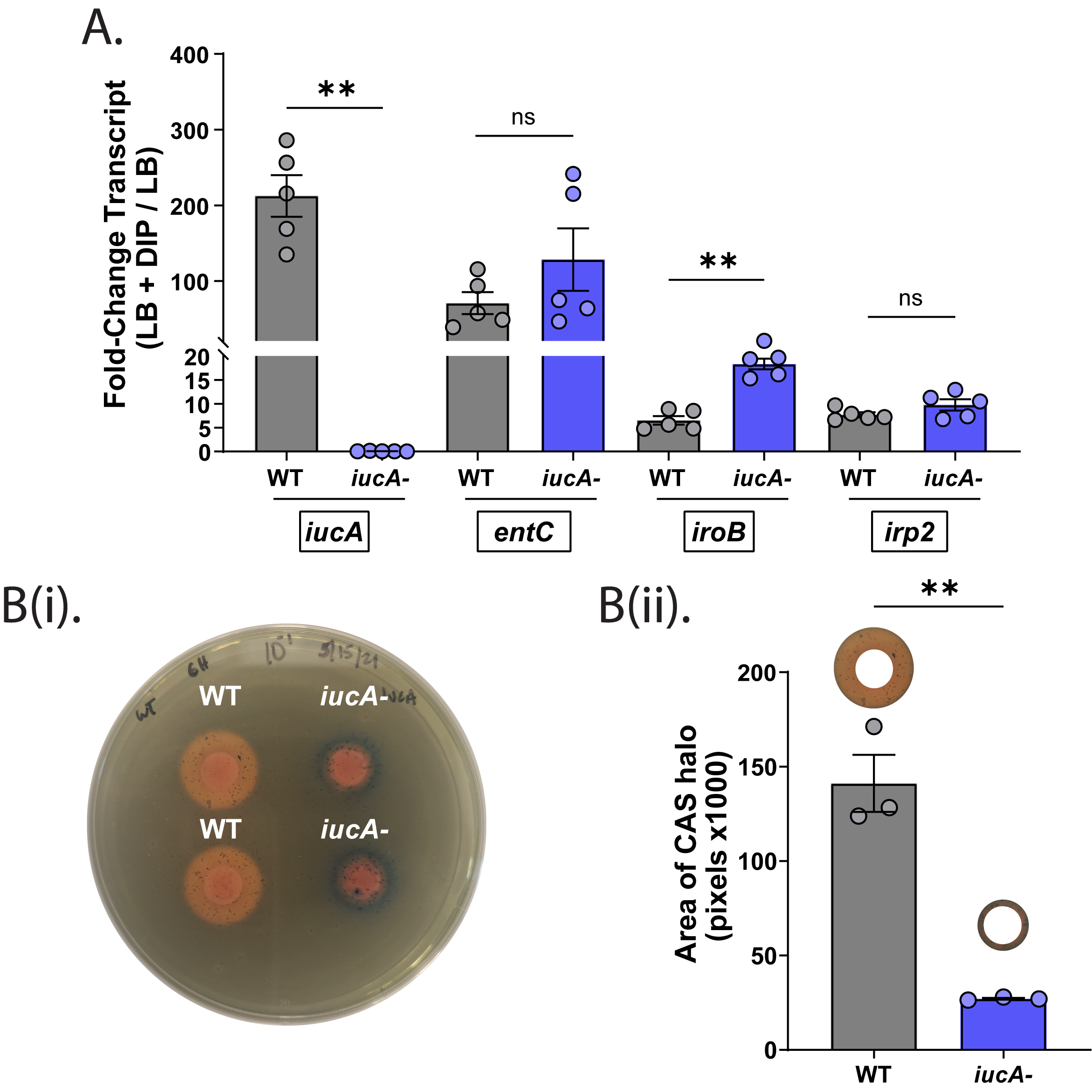

Supplement: S2 Fig — (A) qRT-PCR comparing gene expression of hvKP1 siderophores: aerobactin (iucA), enterobactin (entC), salmochelin (iroB), and yersiniabactin (irp2) in the WT and the iucA- strain grown in either LB + 200 µM DIP (iron-chelated media) or LB (iron-replete media) at 37°C under aerobic conditions. For qRT-PCR, each biological replicate (n = 5) was run as duplicates. Shown is the fold-change in transcription with gyrA used as the internal control for 2-ΔΔCT. (B) Total siderophore production was assessed on CAS agar plates (i) for the WT and the iucA- mutant grown in LB overnight and diluted 1:10 in PBS before plating and incubated for 16 hours at 37°C. Halos were measured and expressed as the mean area in pixels x1000. (ii). Images of representative halos (from (i)) are shown above each bar graph. Statistical significance was calculated using the Mann-Whitney U tests. *P < 0.05, **P < 0.01, ***P < 0.001, and ****P < 0.0001; ns, not significant. (TIF) [file ppat.1014122.s002.tif]

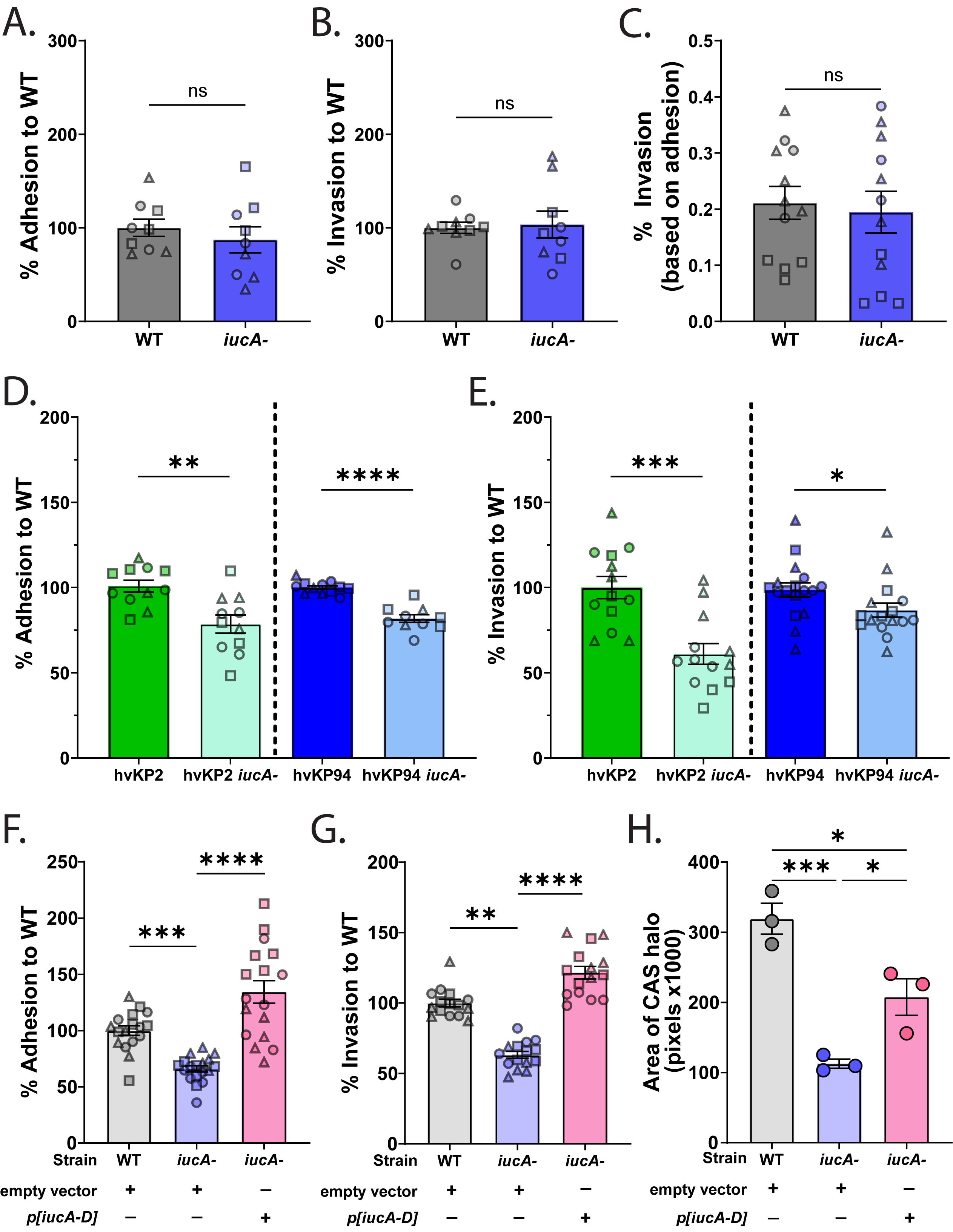

Supplement: S3 Fig — (A-B) Caco-2 monolayers were infected with either the WT (gray) or the iucA- strain (blue) grown in LB (non-chelated media) to mid-log phase (OD600 ~ 0.5) at 37°C under aerobic conditions to test for adhesion (A) or invasion (B) at an MOI of 50. (C) Percent Invasion of Caco-2 monolayers infected with either the WT (gray) or the iucA- mutant (blue) grown in LB + 200 µM DIP (iron-chelated media) at an MOI of 50 normalized to adhesion. (D-E) Caco-2 monolayers were infected at an MOI of 50 with either the hvKP2 (green) or hvKP94 (blue) along with their respective iucA- mutants to test for adhesion (D) and invasion (E). Strains were grown in LB + 200 µM DIP (iron-chelated media) to mid-log phase (OD600 ~ 0.5) at 37°C under aerobic conditions, prior to infection. (F-G) Caco-2 monolayers were infected with either the WT containing the empty pFUS2 vector (gray), iucA- with empty vector (blue), or iucA- + pFUS2[iucA-D] complement plasmid (p[iucA-D]) (pink) grown in LB + 200 µM DIP to mid-log phase (OD600 ~ 0.5) at 37°C under aerobic conditions and tested for adhesion (F) and invasion (G) at an MOI of 50. (A-G) The results from 3 independent assays, each with ≥3 replicates are shown, and the data is expressed as % to WT with ±SEM. Statistical significance was calculated using the Mann-Whitney U test when comparing 2 groups or Kruskal-Wallis followed by post-hoc Dunn’s test of multiple comparisons when comparing 3 groups. (H) Total siderophore production was assessed on CAS agar plates for either the WT containing the empty pFUS2 vector (gray), iucA- with empty vector (blue), or iucA- + pFUS2[iucA-D] complement plasmid (p[iucA-D]) (pink). Each strain was grown overnight in LB supplemented with gentamicin (10 µg/mL) and diluted 1:10 in PBS before plating. Halos were measured and expressed as the mean area in pixels x1000. Shown are the averages for each experiment (n = 3). Statistical significance was determined using a one-way ANOVA followed by Tukey’s post-hoc test. *P < 0.05 [file ppat.1014122.s003.tif]

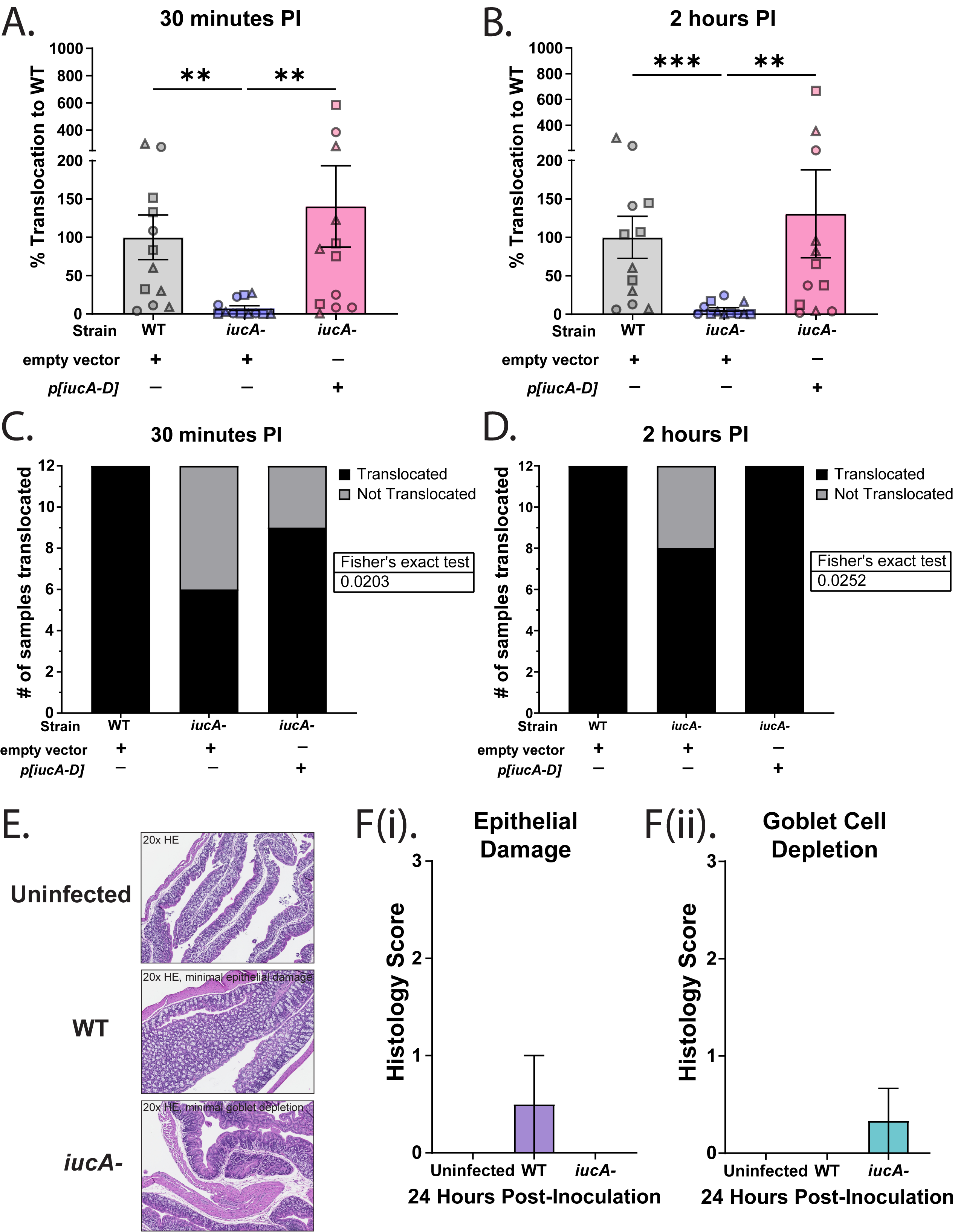

Supplement: S4 Fig — (A-B) Polarized Caco-2 cells were grown on transwell inserts and allowed to differentiate for 19 days before infecting with either the WT containing the empty pFUS2 vector (gray), iucA- with empty vector (blue), or iucA- + pFUS2[iucA-D] complement plasmid (p[iucA-D]) (pink) grown in LB + 200 µM DIP + 5µg/mL gentamicin to mid-log phase (OD600 ~ 0.5) at 37°C under aerobic conditions and tested for translocation. The top and bottom chambers were sampled 30 min PI (A) and 2 hours PI (B) to measure for translocation with data expressed as percent to WT. Shown are the results from 3 independent assays (n = 4) and a Kruskal-Wallis followed by post-hoc Dunn’s test of multiple comparisons was performed for statistical significance. (C-D) Contingency plots (3x2) displaying the translocation outcome for each strain at 30 min PI (C) and 2 hours PI (D). The Fisher’s exact test was performed for statistical significance and the resulting p-values are indicated. (F-G) C57BL6/J mice were colonized with WT, iucA-, or given 2% sucrose PBS-vehicle control (uninfected) (n ≥ 2 per group). Murine proximal colons were collected 24h PI. (F) Representative images for each group of the H&E stained proximal colons (5 µm sections). (G). Blinded histopathology scoring of H&E stained samples for epithelial damage (i) and goblet cell depletion (ii).PI, post-inoculation. **P < 0.01, ***P < 0.001, and ****P < 0.0001; ns, not significant. (TIF) [file ppat.1014122.s004.tif]

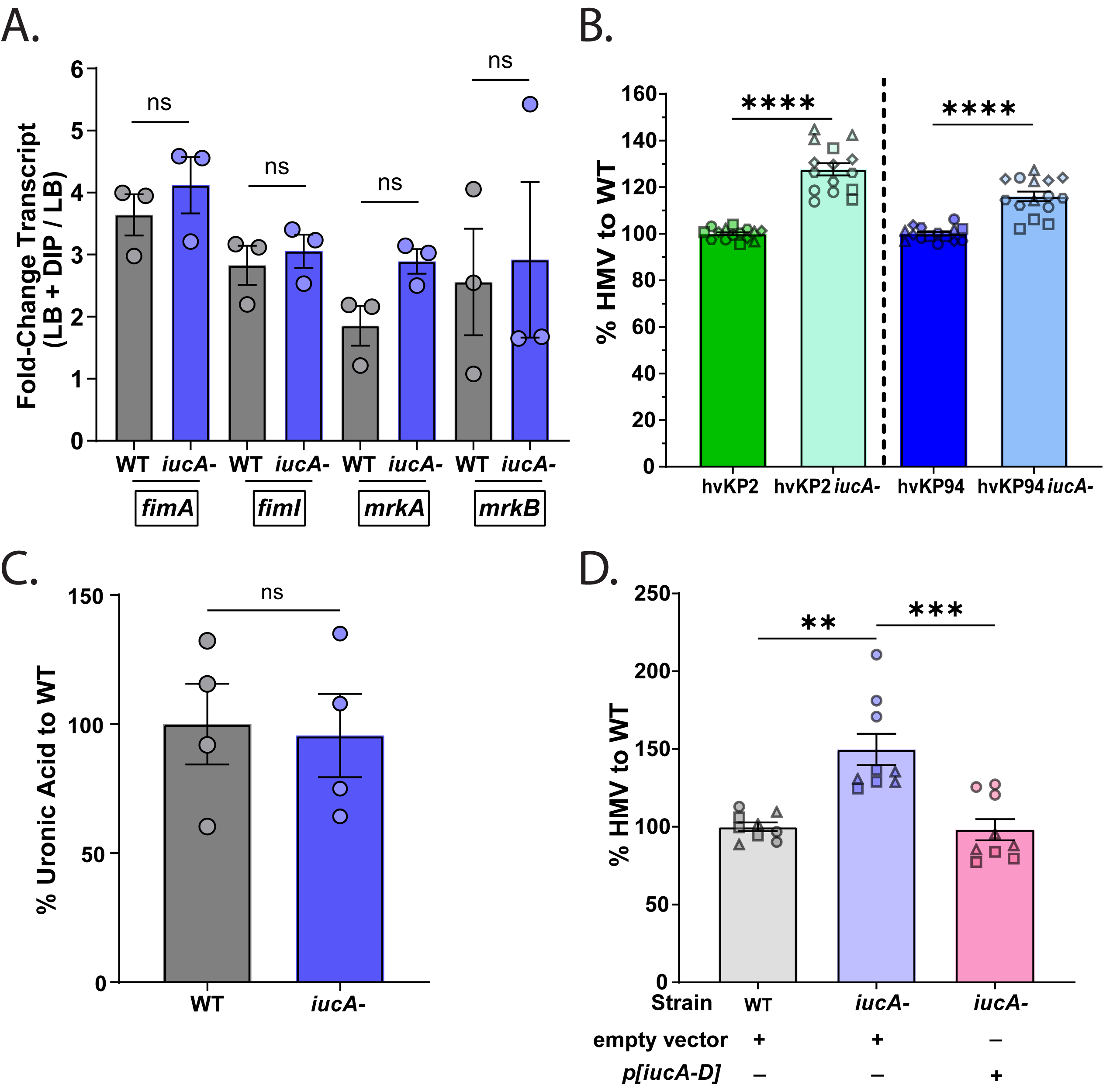

Supplement: S5 Fig — (A) qRT-PCR comparing gene expression of the type 1 (fimA and fimI) and type 3 (mrkA and mrkB) fimbriae genes in the WT and the iucA- strain grown in LB + 200 µM DIP and LB. Each biological replicate (n = 3) was run as duplicates. Shown is the fold-change in transcription with gyrA as the internal control for 2-ΔΔCT analysis. (B) HMV comparison via low-speed spin-down assay of the hvKP2 (green) and hvKP94 (blue) to its respective iucA- isogenic mutant grown to mid-log phase (OD600 ~ 0.5) in LB + 200 µM DIP (iron-chelated media), and expressed as the percent to WT. Shown is the average of 5 independent assays each performed in triplicate. Mann-Whitney U tests were performed to determine statistical significance. (C) Uronic acid content was quantified as a metric of capsule production for the WT (gray) and the iucA- mutant (blue) grown to mid-log phase (OD600 ~ 0.5) in iron-chelated media (LB + 200 µM DIP) at 37°C under aerobic conditions. Mann-Whitney U tests were performed to determine statistical relevance. Shown are the averages of 4 independent assays, each with 3 replicates. (D) HMV comparison of the WT containing the pFus2 empty vector (gray), iucA- + empty vector (blue), and iucA- + p[iucA-D] (pink) grown to mid-log phase (OD600 ~ 0.5) in LB + 200 µM DIP at 37°C under aerobic conditions and expressed as percent to WT. The results of 3 independent assays, each with 3 replicates are shown and a Kruskal-Wallis followed by post-hoc Dunn’s test of multiple comparisons was performed to determine statistical significance. *P < 0.05, **P < 0.01, ***P < 0.001, and ****P < 0.0001; ns, not significant. (TIF) [file ppat.1014122.s005.tif]
