# Supplementary material for: Aerobactin is a key driver of hypervirulent Klebsiella pneumoniae translocation and virulence
Source: PLoS Pathog. 2026 Apr 13;22(4):e1014122. doi: 10.1371/journal.ppat.1014122 (PMC13089870; doi:10.1371/journal.ppat.1014122)
Supplement: S1 Table — (DOCX) [file ppat.1014122.s006.docx]

| **Strain ID** | **Description** | **Resistance** | **Siderophore/Genotoxin locus*** | **Reference** |
| --- | --- | --- | --- | --- |
| AZ71 | hvKP1 (ST86, K2 serotype) clinical isolate | Amp^r^ | *ent^+^, iuc^+^, iro^+^, irp^+^* | (1) |
| AZ72 | hvKP2 (K1 serotype) clinical isolate | Amp^r^ | *ent^+^, iuc^+^, iro^+^, irp^+^, clb^+^* | (2) |
| AZ74 | hvKP1 *iucA::kan* | Amp^r^, Kan^r^ | *-* | (3) |
| AZ77 | hvKP1 *entB::kan, irp2::hyg* | Amp^r^, Kan^r^, Hyg^r^ | - | (4) |
| AZ94 | KPPR1 (ATCC43816), *att::apra* | Amp^r^, Rif^r^, Apr^r^ | *ent^+^, iro^+^, irp^+^* | (5) |
| AZ165 | hvKP94 (K2 serotype) clinical isolate | Amp^r^ | *ent^+^, iuc^+^, iro^+^, irp^+^* | (6) |
| AZ234 | hvKP2 *iucA::kan* | Amp^r^, Kan^r^ | - | This study |
| AZ255 | hvKP94 *iucA::kan* | Amp^r^, Kan^r^ | - | This study |
| AZ265 | hvKP1 *ΔiucA* | Amp^r^ | - | This study |
| AZ314 | hvKP1 *attTn7::apra* | Amp^r^, Apr^r^ | - | This study |
| AZ325 | hvKP1 *ΔiucA, irp2::kan* | Amp^r^, Kan^r^ | - | This study |
| AZ329 | hvKP1 *ΔiucA, irp2::kan, entB::cam* | Amp^r^, Kan^r^, Cam^r^ | - | This study |
| AZ349 | hvKP1 *rmpD::kan* | Amp^r^, Kan^r^ | - | This study |
| AZ350 | hvKP1 *ΔiucA, rmpD::kan* | Amp^r^, Kan^r^ | - | This study |

**S1 Table. Strains used in the study**

****ent, enterobactin;iuc, aerobactin; iro, salmochelin; irp, yersiniabactin; clb, colibactin.***

1. Pomakova DK, Hsiao CB, Beanan JM, Olson R, MacDonald U, Keynan Y, et al. Clinical and phenotypic differences between classic and hypervirulent Klebsiella pneumonia: an emerging and under-recognized pathogenic variant. Eur J Clin Microbiol Infect Dis. 2012;31(6):981-9.

2. Russo TA, Olson R, Fang CT, Stoesser N, Miller M, MacDonald U, et al. Identification of Biomarkers for Differentiation of Hypervirulent Klebsiella pneumoniae from Classical K. pneumoniae. J Clin Microbiol. 2018;56(9).

3. Russo TA, Olson R, Macdonald U, Metzger D, Maltese LM, Drake EJ, et al. Aerobactin mediates virulence and accounts for increased siderophore production under iron-limiting conditions by hypervirulent (hypermucoviscous) Klebsiella pneumoniae. Infect Immun. 2014;82(6):2356-67.

4. Russo TA, Olson R, MacDonald U, Beanan J, Davidson BA. Aerobactin, but not yersiniabactin, salmochelin, or enterobactin, enables the growth/survival of hypervirulent (hypermucoviscous) Klebsiella pneumoniae ex vivo and in vivo. Infect Immun. 2015;83(8):3325-33.

5. Agard MJ, Ozer EA, Morris AR, Piseaux R, Hauser AR. A Genomic Approach To Identify Klebsiella pneumoniae and Acinetobacter baumannii Strains with Enhanced Competitive Fitness in the Lungs during Multistrain Pneumonia. 2019;87(6):10.1128/iai.00871-18.

6. Russo TA, MacDonald U, Hassan S, Camanzo E, LeBreton F, Corey B, et al. An Assessment of Siderophore Production, Mucoviscosity, and Mouse Infection Models for Defining the Virulence Spectrum of Hypervirulent Klebsiella pneumoniae. mSphere. 2021;6(2).
