# Supplementary material for: Aerobactin is a key driver of hypervirulent Klebsiella pneumoniae translocation and virulence
Source: PLoS Pathog. 2026 Apr 13;22(4):e1014122. doi: 10.1371/journal.ppat.1014122 (PMC13089870; doi:10.1371/journal.ppat.1014122)
Supplement: S2 Table — (DOCX) [file ppat.1014122.s007.docx]

**S2 Table. Plasmids used in the study**

| **Plasmid** | **Description** | **Antibiotic Marker** | **Reference** |
| --- | --- | --- | --- |
| pKD46 | λ red recombinase genes in pBAD vector | Spc^r^ | (1) |
| pKD4 | Kanamycin cassette with FRT sites | Kan^r^ | (1) |
| pFlp3 | Flp recombinase for removing FRT *kan* cassette | Amp^r^, Tet^r^ | (2) |
| pFUS2 | *oriV*^ColE1^ *oriT*^RK2^ *lacZ* transcriptional reporter; suicide vector, Gmr | Gen^r^ | (3) |
| pFUS2[iucA-D] | pFUS2 expressing the full-length *iuc* operon | Gen^r^ | (4) |

1. Datsenko KA, Wanner BL. One-step inactivation of chromosomal genes in Escherichia coli K-12 using PCR products. 2000;97(12):6640-5.

2. Choi K-H, Gaynor JB, White KG, Lopez C, Bosio CM, Karkhoff-Schweizer RR, et al. A Tn7-based broad-range bacterial cloning and expression system. Nature Methods. 2005;2:443+.

3. Antoine R, Alonso S, Raze D, Coutte L, Lesjean S, Willery E, et al. New Virulence-Activated and Virulence-Repressed Genes Identified by Systematic Gene Inactivation and Generation of Transcriptional Fusions in Bordetella pertussis. 2000;182(20):5902-5.

4. Russo TA, Olson R, Macdonald U, Metzger D, Maltese LM, Drake EJ, et al. Aerobactin mediates virulence and accounts for increased siderophore production under iron-limiting conditions by hypervirulent (hypermucoviscous) Klebsiella pneumoniae. Infect Immun. 2014;82(6):2356-67.
