# Supplementary material for: Aerobactin is a key driver of hypervirulent Klebsiella pneumoniae translocation and virulence
Source: PLoS Pathog. 2026 Apr 13;22(4):e1014122. doi: 10.1371/journal.ppat.1014122 (PMC13089870; doi:10.1371/journal.ppat.1014122)
Supplement: S3 Table — (DOCX) [file ppat.1014122.s008.docx]

**S3 Table. Primers used in the study**

| **Primer Name** | **Description** | **Orientation** | **Sequence (5’  3’)** |
| --- | --- | --- | --- |
| iucA-US-F | Upstream *iucA* gene | Forward | TTTTGCCCGACACCGAGTAA |
| iucA-DS-R | Downstream *iucA* gene | Reverse | AAATATCTGCTCCAGCGCGT |
| iucA-pKD4-F | Amplifying kanR cassette from pKD4 for knocking out *iucA* | Forward | GTGCTTATTTATAAAATAAATTTTATCAATCCTAATAATTTTTGATGATAtgtgtaggctggagctgcttc |
| iucA-pKD4-R | Amplifying kanR cassette from pKD4 for knocking out *iucA* | Reverse | CAGCGCAGTCCATATCCGCTGTGAACGATGTTTGCCTTAGACATTTAACCcatgggaattagccatggtcc |
| Kan-Forward K1 | Sequence of kanR cassette from pKD4 to confirm insertion | Forward | GGGCACAACAGACAATCGGC |
| Kan-Reverse-K2 | Sequence of kanR cassette from pKD4 to confirm insertion | Reverse | GCAGTTCATTCAGGGCACCG |
| irp2-US-F | Upstream *irp2* gene | Forward | CCTTTAAACGCGTGATGTAACC |
| irp2-DS-R | Downstream *irp2* gene | Reverse | CACGACCTTCCAGCAGATTT |
| irp2-pKD4-F | Amplifying kanR cassette from pKD4 for knocking out *irp2* | Forward | TATTCTCATATGAGCAATGCTTTTCGGTAAGACGTGCCATCAGGAGGAAGAATGATTTCTtgtgtaggctggagctgcttc |
| irp2-pKD4-R | Amplifying kanR cassette from pKD4 for knocking out *irp2* | Reverse | GAACGCCATGCGTTCGCGATGGCGTTCCGGGGAAAATCAGTTTGCTTCGCGCTATATCCGcatgggaattagccatggtcc |
| entB_US-2-F | Amplifying *entB::Cam* cassette from MKP103 transposon library | Forward | ACTATGCGATGAGTTCCCCG |
| entB_DS-2-R | Amplifying *entB::Cam* cassette from MKP103 transposon library | Reverse | GCTTAAACTGCTCGCCGAAG |
| rmpA-int-F | Upstream *rmpD* gene | Forward | TTCAGGGAAATGGGGAGGGTA |
| rmpC-int-R | Downstream *rmpD* gene | Reverse | CTCGCGTGCGTTAATGATGT |
| rmpD-pKD4-US | Amplifying kanR cassette from pKD4 for knocking out *rmpD* | Forward | ATGAAAGATGGCTCATGCCAAGTATTTAGGTAAAAAAGGGGAGGGGATGTGAAGGATCTCtgtgtaggctggagctgcttc |
| rmpD-pKD4-DS | Amplifying kanR cassette from pKD4 for knocking out *rmpD* | Reverse | GGCATGAGTTATATATCGCGCTGATATCATTTATTGAACGTGAGTATAGTTCTATAATAGgcatatgaatatcctcctta |
| GlmS Crepin FWD | Confirm insertion of kanR cassette at *attTn7* and used to amplify *attTn7::apra* from AZ94 | Forward | ACATGCACATCATTGAGATGCCGC |
| pstS Crepin REV | Confirm insertion of kanR cassette at *attTn7* and used to amplify *attTn7::apra* from AZ94 | Reverse | ATCTGCTTAACGCCACCAGAGGAA |
| Kpn-16S-Long-F | qRT-PCR primer for *K. pneumoniae* 16S | Forward | GGTTGTAAAGCACTTTGAGCGG |
| Kpn-16S-Long-R | qRT-PCR primer for *K. pneumoniae* 16S | Reverse | GAGGTCGCTTCTCTTTGTGTATGC |
| q-ivgyrA1-F | qRT-PCR primer for *K. pneumoniae* gyrA | Forward | TCGGCATCAACATGGTAGCT |
| q-ivgyrA1-R | qRT-PCR primer for *K. pneumoniae* gyrA | Reverse | ACGCGGCGATGATGTCC |
| rpoD-F | qRT-PCR primer for *K. pneumoniae* *rpoD* | Forward | GATCTGATCACCGGTTTCGT |
| rpoD-R | qRT-PCR primer for *K. pneumoniae* *rpoD* | Reverse | CTTCGTCGTCATCCATCTCTTC |
| q-iucA-F | qRT-PCR primer for *K. pneumoniae* *iucA* | Forward | GCGTGATCAGGTGACTCATAAA |
| q-iucA-R | qRT-PCR primer for *K. pneumoniae iucA* | Reverse | CTCGTTGTGGTCGTTCAGATAA |
| q-entC-F | qRT-PCR primer for *K. pneumoniae entC* | Forward | CGACAAAGTGGTGCTATCAAGG |
| q-entC-R | qRT-PCR primer for *K. pneumoniae entC* | Reverse | GCGGCACGTGGAAGTTAAA |
| q-iroB-F | qRT-PCR primer for *K. pneumoniae iroB* | Forward | GTAAACGTCTGCTGGTGAGT |
| q-iroB-R | qRT-PCR primer for *K. pneumoniae iroB* | Reverse | GCCGGAAGATGCAGGATAAT |
| q-irp2-F | qRT-PCR primer for *K. pneumoniae irp2* | Forward | CTGATGAACTCACTCGCTATCC |
| q-irp2-R | qRT-PCR primer for *K. pneumoniae irp2* | Reverse | GCACAGACCTGATAACCTTCA |
| rmpA-F | qRT-PCR primer for *K. pneumoniae rmpA* | Forward | AGTTAACTGGACTACCTCTGTTTC |
| rmpA-R | qRT-PCR primer for *K. pneumoniae rmpA* | Reverse | TCCTGCAGTCAACCAATACTC |
| rmpD-F | qRT-PCR primer for *K. pneumoniae rmpD* | Forward | GCACTCTTTCATTTATTTATTTTTTTATTTCTG |
| rmpD-R | qRT-PCR primer for *K. pneumoniae rmpD* | Reverse | CGTCTATTTATCAATGTTCTGTGC |
| rmpC-F | qRT-PCR primer for *K. pneumoniae rmpC* | Forward | CATGCACTCCTGATTCAAACG |
| rmpC-R | qRT-PCR primer for *K. pneumoniae rmpC* | Reverse | TCCGATGAGGGTGGAATTAAC |
| q-manC-F | qRT-PCR primer for *K. pneumoniae manC* | Forward | CAGCGGCATGTTTATGTTCC |
| q-manC-R | qRT-PCR primer for *K. pneumoniae manC* | Reverse | ATGAAGTCGCTGCCGTTAT |
| q-galF-F | qRT-PCR primer for *K. pneumoniae galF* | Forward | TGCTGCCGATCGTTGATAAG |
| q-galF-R | qRT-PCR primer for *K. pneumoniae galF* | Reverse | GTAGGATGTGTCGAAGTGGTTT |
| q-wzi-F | qRT-PCR primer for *K. pneumoniae galF* | Forward | ACAGATAACGAACCGGGTAAC |
| q-wzi-R | qRT-PCR primer for *K. pneumoniae galF* | Reverse | CCAACCATCTGCCCATAGAA |
| fimA-F | qRT-PCR primer for *K. pneumoniae fimA* | Forward | ATTGTTGTGTCAGCCCTGT |
| fimA-R | qRT-PCR primer for *K. pneumoniae fimA* | Reverse | CCGCATTAACGACTTCTCCT |
| FimI-F | qRT-PCR primer for *K. pneumoniae fimI* | Forward | CTGATAATGGCGAGCTCCTG |
| FimI-R | qRT-PCR primer for *K. pneumoniae fimI* | Reverse | TTCGCCGACAGCATGAAA |
| mrkA-F | qRT-PCR primer for *K. pneumoniae mrkA* | Forward | TAAGCAAACTGGGCGTGAA |
| mrkA-R | qRT-PCR primer for *K. pneumoniae mrkA* | Reverse | TAGCCCTGTTGTTTGCTGGT |
| mrkB-F | qRT-PCR primer for *K. pneumoniae mrkB* | Forward | TGTTAACGGTACCCGCTTTATTT |
| mrkb-R | qRT-PCR primer for *K. pneumoniae mrkB* | Reverse | GGACGGTCGGCGTTATTT |

UPPERCASE: bacteria-specific sequence; lowercase: plasmid-specific sequence
