# Supplementary material for: Aerobactin is a key driver of hypervirulent Klebsiella pneumoniae translocation and virulence
Source: PLoS Pathog. 2026 Apr 13;22(4):e1014122. doi: 10.1371/journal.ppat.1014122 (PMC13089870; doi:10.1371/journal.ppat.1014122)
Supplement: Attachment — Submitted filename: Response to Reviewers.docx [file ppat.1014122.s011.docx]

PPATHOGENS-D-25-01441
Aerobactin is a key driver of hypervirulent *Klebsiella pneumoniae* translocation and virulence
PLOS Pathogens

Dear Dr. Zafar,

Thank you for submitting your manuscript to PLOS Pathogens. After careful consideration, we feel that it has merit but does not fully meet PLOS Pathogens's publication criteria as it currently stands. Therefore, we invite you to submit a revised version of the manuscript that addresses the points raised during the review process.

Points raised by multiple reviewers and to be addressed in a revised manuscript are genetic complementation studies and epithelial cell translocation assays. Please submit your revised manuscript within 60 days Oct 30 2025 11:59PM. If you will need more time than this to complete your revisions, please reply to this message or contact the journal office at [plospathogens@plos.org](mailto:plospathogens@plos.org). When you're ready to submit your revision, log on to [https://www.editorialmanager.com/ppathogens/](https://urldefense.com/v3/__https:/www.editorialmanager.com/ppathogens/__;!!Kv7QgGdTlhIaqSqT!MFuTqxE46KKc6cuFFDLxZvChgamOMZhHDGTG5pwXmVFg59HMotCBpZx03gFJ800zuGflZgxlFKxGDgr_8yKusOqG9g$) and select the 'Submissions Needing Revision' folder to locate your manuscript file.

Please include the following items when submitting your revised manuscript:

* A rebuttal letter that responds to each point raised by the editor and reviewer(s). You should upload this letter as a separate file labeled 'Response to Reviewers'. This file does not need to include responses to any formatting updates and technical items listed in the 'Journal Requirements' section below.
* A marked-up copy of your manuscript that highlights changes made to the original version. You should upload this as a separate file labeled 'Revised Manuscript with Track Changes'.
* An unmarked version of your revised paper without tracked changes. You should upload this as a separate file labeled 'Manuscript'.

If you would like to make changes to your financial disclosure, competing interests statement, or data availability statement, please make these updates within the submission form at the time of resubmission. Guidelines for resubmitting your figure files are available below the reviewer comments at the end of this letter.

We look forward to receiving your revised manuscript.

Kind regards,

Leigh Knodler
Academic Editor
PLOS Pathogens

Thomas Guillard
Section Editor
PLOS Pathogens

Sumita Bhaduri-McIntosh
Editor-in-Chief
PLOS Pathogens
orcid.org/0000-0003-2946-9497

Michael Malim
Editor-in-Chief
PLOS Pathogens
orcid.org/0000-0002-7699-2064


**Journal Requirements:**

1) We ask that a manuscript source file is provided at Revision. Please upload your manuscript file as a .doc, .docx, .rtf or .tex. If you are providing a .tex file, please upload it under the item type u2018LaTeX Source Fileu2019 and leave your .pdf version as the item type u2018Manuscriptu2019.

2) We do not publish any copyright or trademark symbols that usually accompany proprietary names, eg ©,  ®, or TM  (e.g. next to drug or reagent names). Therefore please remove all instances of trademark/copyright symbols throughout the text, including:
- ® on pages: 19, 23, and 39
- TM on pages: 20, 21, 22, and 23.

3) Please upload all main figures as separate Figure files in .tif or .eps format. For more information about how to convert and format your figure files please see our guidelines: 
[https://journals.plos.org/plospathogens/s/figures](https://urldefense.com/v3/__https:/journals.plos.org/plospathogens/s/figures__;!!Kv7QgGdTlhIaqSqT!MFuTqxE46KKc6cuFFDLxZvChgamOMZhHDGTG5pwXmVFg59HMotCBpZx03gFJ800zuGflZgxlFKxGDgr_8yIH2JeHJA$)

4) We have noticed that you have uploaded Supporting Information files, but you have not included a list of legends. Please add a full list of legends for your Supporting Information files after the references list.

5) Some material included in your submission may be copyrighted. According to PLOSu2019s copyright policy, authors who use figures or other material (e.g., graphics, clipart, maps) from another author or copyright holder must demonstrate or obtain permission to publish this material under the Creative Commons Attribution 4.0 International (CC BY 4.0) License used by PLOS journals. Please closely review the details of PLOSu2019s copyright requirements here: PLOS Licenses and Copyright. If you need to request permissions from a copyright holder, you may use PLOS's Copyright Content Permission form.
Please respond directly to this email and provide any known details concerning your material's license terms and permissions required for reuse, even if you have not yet obtained copyright permissions or are unsure of your material's copyright compatibility. Once you have responded and addressed all other outstanding technical requirements, you may resubmit your manuscript within Editorial Manager. 
Potential Copyright Issues:
i) Figures 1B, and 2A. Please confirm whether you drew the images / clip-art within the figure panels by hand. If you did not draw the images, please provide (a) a link to the source of the images or icons and their license / terms of use; or (b) written permission from the copyright holder to publish the images or icons under our CC BY 4.0 license. Alternatively, you may replace the images with open source alternatives. See these open source resources you may use to replace images / clip-art:
- [https://commons.wikimedia.org](https://urldefense.com/v3/__https:/commons.wikimedia.org__;!!Kv7QgGdTlhIaqSqT!MFuTqxE46KKc6cuFFDLxZvChgamOMZhHDGTG5pwXmVFg59HMotCBpZx03gFJ800zuGflZgxlFKxGDgr_8yKP0QIgTA$)
- [https://openclipart.org/](https://urldefense.com/v3/__https:/openclipart.org/__;!!Kv7QgGdTlhIaqSqT!MFuTqxE46KKc6cuFFDLxZvChgamOMZhHDGTG5pwXmVFg59HMotCBpZx03gFJ800zuGflZgxlFKxGDgr_8yIelONcmw$).

Downloaded images from SciDraw and edited them on Inkscape and Adobe illustrator

1B) Bone scissors, Repeating pipettor, Mouse at different ages, – all downloaded 5/29/24

2A) Same mouse and tools from 1B but downloaded 1mL syringe with 25G needed

<https://beta.scidraw.io/drawing/461>

**copied**

**Scidraw**, coded with love by [**Federico and Alex**](https://scidraw.io/contact/).

[**@scidrawio**](https://twitter.com/scidrawio)

Thank you to [**everyone**](https://scidraw.io/contributors/) who uploaded drawings to **Scidraw**.

Scidraw is supported by the [**Sainsbury Wellcome Centre**](https://www.sainsburywellcome.org/web/).

All content on SciDraw is shared under creative commons lincense ([**CC-BY**](https://creativecommons.org/licenses/by/4.0/)) unless stated otherwise. [**Terms and Conditions**](https://scidraw.io/terms/)

Downloaded tweezer for 1B from Bioicons 6/6/24 and modified on inkscape and illustrator

tweezer icon by Servier https://smart.servier.com/ is licensed under CC-BY 3.0 Unported https://creativecommons.org/licenses/by/3.0/

6) We note that your Data Availability Statement is currently as follows: "All data supporting the findings of this study are available within the manuscript and its supporting information files.". Please confirm at this time whether or not your submission contains all raw data required to replicate the results of your study. Authors must share the “minimal data set” for their submission. PLOS defines the minimal data set to consist of the data required to replicate all study findings reported in the article, as well as related metadata and methods ([https://journals.plos.org/plosone/s/data-availability#loc-minimal-data-set-definition](https://urldefense.com/v3/__https:/journals.plos.org/plosone/s/data-availability*loc-minimal-data-set-definition__;Iw!!Kv7QgGdTlhIaqSqT!MFuTqxE46KKc6cuFFDLxZvChgamOMZhHDGTG5pwXmVFg59HMotCBpZx03gFJ800zuGflZgxlFKxGDgr_8yKOqFCKPQ$)).
For example, authors should submit the following data: 
1) The values behind the means, standard deviations and other measures reported;
2) The values used to build graphs;
3) The points extracted from images for analysis..
Authors do not need to submit their entire data set if only a portion of the data was used in the reported study.
If your submission does not contain these data, please either upload them as Supporting Information files or deposit them to a stable, public repository and provide us with the relevant URLs, DOIs, or accession numbers. For a list of recommended repositories, please see [https://journals.plos.org/plosone/s/recommended-repositories](https://urldefense.com/v3/__https:/journals.plos.org/plosone/s/recommended-repositories__;!!Kv7QgGdTlhIaqSqT!MFuTqxE46KKc6cuFFDLxZvChgamOMZhHDGTG5pwXmVFg59HMotCBpZx03gFJ800zuGflZgxlFKxGDgr_8yIhHCV64A$). 
If there are ethical or legal restrictions on sharing a de-identified data set, please explain them in detail (e.g., data contain potentially sensitive information, data are owned by a third-party organization, etc.) and who has imposed them (e.g., an ethics committee). Please also provide contact information for a data access committee, ethics committee, or other institutional body to which data requests may be sent. If data are owned by a third party, please indicate how others may request data access.

7) Please amend your detailed Financial Disclosure statement. This is published with the article. It must therefore be completed in full sentences and contain the exact wording you wish to be published.
1) State the initials, alongside each funding source, of each author to receive each grant. For example: "This work was supported by the National Institutes of Health (####### to AM; ###### to CJ) and the National Science Foundation (###### to AM)."
2) State what role the funders took in the study. If the funders had no role in your study, please state: "The funders had no role in study design, data collection and analysis, decision to publish, or preparation of the manuscript."
3) If any authors received a salary from any of your funders, please state which authors and which funders..

Note: If the reviewer comments include a recommendation to cite specific previously published works, please review and evaluate these publications to determine whether they are relevant and should be cited. There is no requirement to cite these works unless the editor has indicated otherwise.


**Reviewers' Comments:**

Reviewer's Responses to Questions

**Part I - Summary**
Please use this section to discuss strengths/weaknesses of study, novelty/significance, general execution and scholarship.

**Reviewer #1:** In this manuscript by Hernandez and colleagues, the authors seek to understand the role of aerobactin in the colonization and translocation of hypervirulent Klebsiella pneumoniae strains from the gut to sterile sites. Using in vitro and in vivo approaches, the authors define that aerobactin is responsible for Kp translocation in the gut niche to sterile sites and for overall Kp pathogenicity during infection. The experiments are well controlled, analyzed, and interpreted. My main concern is the lack of complementation and contextualization within the Kp pathogenesis field. The authors do not cite literature that is relevant to the study, which lessens the overall impact of the work. There is also a lack of in vivo complementation data that would support overall findings from this study.

**Reviewer #2:** The paper addresses a key ‘sink’ for acquired drug resistance, hypervirulent Klebsiella pneumoniae (hvKp), and its ability to move from the gut environment to the bloodstream where it can causes sepsis. hvKp present something of enigma insofar as they need to adhere to especially epithelial mucosae, yet often express a mucoid capsule which presents a barrier to adhesion. Once through the epithelial barrier and the iron-rich environment of the gut, they need to acquire iron where much of this key micronutrient is sequestered by host iron-binding proteins. hvKp can have multiple systems for acquiring iron, and express up to 4 siderophore systems. The authors have investigated the link between hvKp virulence, the virulence plasmid expression of aerobactin (iuc genes), gut colonisation in competition with a rich microbiome and specifically translocation through the epithelia. The authors acknowledge that considerable work has been published on closely related studies that used different models for lung and soft tissue infection, and sepsis. The authors make some interesting and important observations that adds to our understanding of siderophores in hvKp.

**Reviewer #3:** Siderophores are important for gut colonization by enteric pathogens, and aerobactin, a siderophore encoded on virulence plasmids commonly found in hypervirulent and convergent K. pneumoniae, has been associated with increased virulence. However, supporting evidence has been largely correlative. This study provides compelling evidence that aerobactin is not essential for gut colonization but plays a critical role in translocation of K. pneumoniae to extraintestinal sites. This finding significantly advances our understanding of K. pneumoniae pathogenesis and may influence future strategies for screening patients colonized with aerobactin-positive hvKp strains. The manuscript is clearly written, well-controlled, and will be of broad interest to the field. I offer a few minor suggestions below to enhance clarity for a general audience.

**Reviewer #4**: In this manuscript entitled "Aerobactin is a key driver of hypervirulent Klebsiella pneumoniae translocation and virulence" by Hernandez et al., the authors investigated the role of aerobactin in facilitating gastrointestinal translocation and systemic dissemination of hypervirulent Klebsiella pneumoniae (hvKP). Using a murine gastrointestinal colonization model with intact microbiota, the authors compared pathogenesis of an hvKP clinical isolate (hvKP1) to an isogenic aerobactin biosynthesis mutant (iucA-). It has been shown previously that in pneumonia, IP or SQ systemic infection models aerobactin plays a major role in virulence for strain hvKP1. In this work the authors are evaluating the role of aerobactin in pathogenesis in a gastrointestinal model and are challenged with discerning the role of aerobactin in the GI tract vs its already established role during a systemic infection.

The study demonstrates that while both strains colonized the GI tract similarly, mice colonized with the iucA- mutant exhibited significantly lower bacterial burdens in extra-intestinal organs and a decrease in mortality. In addition, the authors provided data suggesting that within a systemic infection model (tail vein) iuc- mutants also have a defect in virulence. They provided a variety of cell culture assays that showed reduced adhesion, invasion, and translocation capabilities of the iucA- mutant, with increased hypermucoviscosity (HMV) linked to upregulation of the rmp locus. Overall, this manuscript is very well written, with some strong evidence, iron-homestasis plays a role in regulating surface associated factors and provides substantial data to support a conclusion that iron-homeostasis is complicated and highly important for virulence in K. pneumoniae. This manuscript includes a substantial body of work that is both informative and impactful to the field.

However, many of the authors conclusions are broadly stated where the data does not support them and should be softened to allow for alternate interpretations and more accurate descriptions. The authors' conclusion that this represents a specific translocation defect is questionable, as the in vitro translocation data presented is relatively weak and would benefit from more rigorous experimental validation. While this work attempts to identify a role for aerobactin in bacterial translocation, the experimental evidence supporting translocation-specific effects is insufficient, and the interpretation of comparative extra-intestinal bacterial burden and virulence between GI and systemic models requires more thorough analysis and discussion. The authors do not provide convincing data to support their hypothesis for a role for aerobactin in translocation vs a role for aerobactin in survival and expansion in the blood stream and extraintestinal organs.

**Part II – Major Issues: Key Experiments Required for Acceptance**
Please use this section to detail the key new experiments or modifications of existing experiments that should be absolutely required to validate study conclusions.

Generally, there should be no more than 3 such required experiments or major modifications for a "Major Revision" recommendation. If more than 3 experiments are necessary to validate the study conclusions, then you are encouraged to recommend "Reject".

**Reviewer #1:**

- In Figure S1, I am concerned that the iucA mutant is not actually defective in iron depleted media. There are no competitive defects in direct 1:1 competitions (Figure S1 Bi and Bii) and the CFU counts for Aii and Aiii are within an error range for serial quantification. Due to the data in subsequent figures, I do not think this lessens the impact of the overall story but these data should be reframed to say that mutations in iucA do not have a dramatic effect on growth in iron depleted media but do impact dissemination. Because other siderophores are likely compensating in the absence of iucA and because icuA is impacting hypermucoviscosity, it is likely that pathogenesis effects are due to capsule and not iron uptake. As such, this work may give new insight in to a new role of aerobactin, unrelated to iron uptake, which is still interesting.

**We appreciate the reviewer for their thoughtful comments on our study. We would like to point out here that when the WT and the mutant are grown together (1:1), and we do not expect to observe a growth defect, rather a compensation, because the WT strain is expected to share the synthesized aerobactin siderophore with the mutant, which while unable to synthesize aerobactin still retains the receptor IutA, allowing it to uptake aerobactin, and grow similarly to WT (trans complementation).** **Furthermore, while our studies were performed with LB, which is nutrient rich, where the growth defects are not pronounced, we would like to draw the attention of the reviewer to the study by *Russo et al.* (PMID 24664504), which used the same isolates and demonstrated the defect of *iucA* mutant in more physiologically relevant growth conditions such as urine, ascites fluid and serum.**

- Because iucA mutations may be affecting multiple cellular processes, it is important to complement this phenotype in vivo. The authors should demonstrate that the fitness defects observed in Figure 1E, 1G, and 2D are related to IucA itself and not off target effects.

**We agree with the reviewer that allelic replacement is critical for fulfilling Koch's postulates. To that end, we were unable to successfully complement the strain chromosomally using various genetic techniques. However, we did use the pFUS2(*iucA-D*) plasmid to successfully complement many of the phenotypes that we observed in our in vitro studies, including CAS agar (fig S3H), HMV (fig. S5D), adhesion (Fig S3F), invasion (Fig S3G), and translocation (Fig S4 A-D), where the plasmid complemented the defect of the iucA- strain. We believe this strongly suggests that the phenotypes observed are due to aerobactin and not secondary mutations. Furthermore, the strain has been extensively used in the field and sequenced (PMID24664504).**

**Furthermore, we attempted to test the iucA mutant with the plasmid in vivo to determine whether the plasmid would complement the mutant's defect. However, as shown below, the plasmid was not retained by the iucA- strain under in vivo conditions, preventing us from carrying out complementation studies. Lastly, we have added a caveat section in the discussion, outlining the limitations of our studies (Ln 461-467). Collectively, this addresses the reviewer's primary concern.**

**Figure: The plasmid pFUS2(iucA-D) is not retained *in vivo* by the *iucA*- mutant.** Mice were inoculated with the iucA- strain containing the pFUS2(iucA-D) plasmid, and twenty-four hours later samples were collected and plated on either ampicillin (25 µg/ml) or gentamicin (10 µg/ml).

- The paper does not appropriately contextualize these findings within the field. Previous literature has shown that most gut isolates of Kp are classical and not hypervirulent which should be mentioned. Previous literature has also shown that Kp burden at other primary cites influences dissemination to secondary sites (PMID39824859). A recent TnSeq study showed that iron uptake systems, such as tonB, are dispensable for hypervirulent Kp pathogenesis during bacteremia (PMID37463183). The findings of Figures 1 and 2 should be placed within the context of this study. Lastly, work into capsule has shown interactions between changes in hypermucoviscosity and Kp attachment and invasion (PMID40595687; PMID37610214).

**While we agree with the reviewer that most Kp gut isolates identified in Western countries are classical strains, in Pacific Rim countries hvKP isolates are commonly associated with human carriage in both community and hospital settings (PMIDs 32576652, 36001493). We have added that detail in our manuscript in the discussion section. (Ln 350-351)**

**Secondly, the study mentioned by the reviewer describes the barcoded library constructed in the strain KPPR1 and used to identify bottlenecks at different sites post-lung inoculation. We have now included these new findings within the context of our data in the results section (Ln 203-205).**

**Third, the study referenced by the reviewer PMID37463183 does list ExbB as a fitness determinant for bacteremia. ExbB is a component of the energy transducing Ton system, using proton motive force (pmf) at the cytoplasmic face to support the active transport of iron-siderophore complexes across the other membrane. Additionally, in that screen they identified FecCD (ABC transporter), and VK055_4427 a tonB dependent receptor family protein. Interestingly, none of the siderophore synthesis or uptake systems were identified in the screen.** **We’ve included this result within the context of our study in the results section (Ln 229-232).**

**Lastly, we appreciate the work suggested by the reviewer, which has now been added into the discussion section to provide further context to our results (Ln 454-460)**

**Reviewer #2:**

The authors have made an important observation about the cell biology concerning an aerobactin synthesis mutant of hvKp – mainly that that can’t egress from an infected cell.

This is very interesting and likely important. The apparent redundancy in the KpSC siderophore systems doesn’t appear to impact here and this phenomenon warrants at least a simple microscopy study to examine where in the cells the bacteria reside, and where they are getting ‘stuck’. The key figure is Fig.4C. Using Transwell studies, the authors note that while approximately half the number of iucA- bacteria enter cells, none emerge on the ? basolateral side of the Caco-2 monolayer. This should be supported by microscopy showing differences in cell signalling. There should be plenty of resources to study eg. P13K/AKT. Whatever the cause and effect, this is not reflected by TEER, in gross histology or buy occluding H-scores.

**We appreciate that the reviewer found our manuscript interesting, and of importance.**

**Regarding the different cell culture assays performed in our manuscript (adhesion, invasion and translocation). Those assays are fundamentally different, while adhesion/invasion assays use around ~500,000 cells (monolayer), translocation assays use less cells which have been allowed to differentiate for ~19 days before the assay is performed. Furthermore, the number of bacteria used to infect the cells is at least a log different. For this reason, we cannot make conclusions from one assay to another but rather interpret the data from single assay.**

**The reviewer also suggest microscopy and identifying host pathways that are likely impacted by *Kpn* interactions with Caco 2 cells. However, previous work by *Hsu et al.* (PMID 25452552) showed through confocal fluorescent microscopy that different *Kpn* strains can adhere and invade several cell lines including Caco-2. They further demonstrated through treatment with compounds (nocodazole and cytochalsin D) that cytoskeleton actin and microtubule are required for *Kpn* invasion of epithelial cells. Through treatment with other inhibitors, they were able to show that cell signaling Rho family GTPases and the P13K/Akt pathway is required for Kpn invasion. While we have cited this work in the discussion section (Ln 408-412), we decided to not replicate these studies and have instead focused on bacterial factors that contribute to *Kpn* interactions with epithelial cells.**

Figure 4 – the data as presented does not as compelling as it might be. Why not represent 3-5 replicates X 3 individually as individual sample ‘dots’? It is hard to understand what level of adherence is actually observed. The MOI of 50 is high and centrifugation aids contact. In the methods, the numbers supposedly link to the inoculum (100%?) but the axis suggest it is relative to WT. Could the numbers of each of the wells tell the same story – there should be at least 10 data points for each figure (3-5 replicates X3). Since Adhesion likely precedes gentamicin resistance (i.e. Invasion), a deficiency in adhesion (4A) is likely to lead to a deficiency in invasion (4B), meaning that the deficit is in adhesion. Again 4B could be presented as raw numbers – how many of the 50 X5 X10(5) bacteria are recovered in the gentamicin protection assay.

**We agree with the reviewer about variability observed in cell culture results. This variability is common in cell culture assays, and different studies show results either as pooled data, or just show a representative assay. Based on reviewers’ suggestion we have now included the pooled data from 3-4 runs with 3-5 replicates per run. Replicates from each run are now represented by unique symbols, allowing us to provide complete data from all the assays performed. While we use normalization to the WT to facilitate comparison across experimental runs, we have also added a Supplemental Table (excel file) that contains all the raw values for the assays and murine infection studies performed in our study. This will allow the readers access to the raw data, which shows the proportion of inoculum that adhered or invaded for each strain and replicate. Regarding the MOI of 50, this is a well-established dose for assessing *K. pneumoniae* host-cell interactions, as seen in the work of *Hsu et al.* and others (e.g., PMID 40768400).**

Reviewer #3: none noted

**Reviewer #4:**

Major Comments:
• The authors' interpretation of their systemic infection model results in the context of their gastrointestinal model is insufficient. While no discernible differences were observed between wild-type and iucA- strain bacterial burden in systemic sites at 6- or 24-hours post infection, the iucA- mutant shows a virulence defect at 8 days post infection. This comparison is not appropriate and does not support their conclusion that “These results likely preclude early clearance of the iucA- as the reason for reduced burden in the extra-intestinal sites” for the reasons described below:
o In the tail vein model, the entire 106 CFU inoculum immediately enters the bloodstream and accumulates in filtration organs, which is fundamentally different from the presumably low numbers of bacteria that translocate across the intestinal epithelium over the course of several hours in the GI model. This is evident at both 6 and 24 hours in the systemic model, where the WT and mutant had 107 CFU/gram in the spleen while in the GI model, the WT had 105 CFU/gram at 24 hours, and the mutant had 103 CFU. It is possible that the differences in CFU found in extra-intestinal organs are due to low levels of translocation into the blood stream for both WT and iucA- mutant but the WT is able to survive and expand at higher numbers than the mutant in the blood stream or extra-intestinal organs. This would not be observed in the tail vein model with administration of such a large dose. This possibility seems especially likely because the iucA- mutant has a growth defect in iron-depleted media.
o The virulence defect of the iucA- mutant in the systemic model, while smaller, is nearly as substantial as that observed in the GI colonization model. The authors fail to adequately discuss comparisons between the two models or provide compelling evidence that the virulence defect observed in the GI model is not due to impaired iron acquisition and subsequent poor survival or inadequate expansion in the extra-intestinal space as is hypothesized for the systemic model.
o Since the authors provide evidence of changes to the cell surface (which could affect survival) in the iuc- mutant, their argument would be strengthened by evaluating survival in the following experiments:
♣ Measuring bacterial survival of WT and iucA- strains in human complement serum.
♣ Performing a macrophage uptake/survival assay
o Taken together, these additional experiments could help distinguish a role in translocation from survival as the cause of differences in bacterial burden observed in the extra-intestinal sites.

**We thank the reviewer for that detailed analysis and for suggesting assays to provide more robust conclusions. We agree that there are caveats when comparing gut colonization to tail vein studies. Our tail vein inoculum was based on studies that use either 10^5^-10^7^ CFU for inoculations (PMID 37463183; 34901898) and picked a dose between those two doses. To provide greater context we picked two different time points and multiple sites, which demonstrated similar level of WT and the *iucA* mutant. While we cannot rule out small growth differences accumulating over time which impact virulence. This data does provide a baseline for us to interpret our GI colonization results, and to provide further molecular details we shifted to cell culture assays.**

**Consequently, based on suggestion of the reviewer we performed complement (serum) killing assays, as the complement system is likely one of the first line of host defenses that *Klebsiella* encounters post translocation. Our results show that there is no difference between the WT and the isogenic mutant strain in our complement killing assay (Fig. 4E), which further suggest that the reduced number of translocated bacteria observed of the mutant are not due to host clearance mechanism at an early timepoint, but rather a translocation defect. We thank the reviewer for suggesting this assay as it further strengthens our results.**

• The authors attempted to validate their findings across genetically diverse hvKP isolates by testing hvKP2 (ST23, KL1) and hvKP94 (ST23, KL1) along with their respective aerobactin-deficient mutants. However, conclusions related to aerobactin's impacts on virulence across genetically distinct isolates are difficult to interpret for several reasons. First, genetic relatedness between the strains is not evaluated, making it unclear whether observed differences reflect strain-specific effects rather than conserved mechanisms.

**These strains were chosen based on the differences in KL type, as KL type 1 and 2 are common hvKP isolates, with all of them containing an intact aerobactin locus. Additionally, hvKP94 was picked because of its partial virulence.**

Second, aerobactin deletions in hvKP2 are known to have little to no defect in virulence in subcutaneous or pneumonia models, and thus the non-statistically significant differences found in the GI model are not convincing of an essential role for aerobactin in hvKP2. (DOI: 10.1016/j.ebiom.2024.105302 and unpublished data).

**We agree with the reviewer that hvKP2 aerobactin mutant retains its high virulence in CD1 outbred mice in the subcutaneous model of infection. Our results with hvKP2 demonstrate that its aerobactin mutant colonizes similarly to the WT the GI tract. These results are in line with the two other isolates hvKP1 and 94, where the mutant colonizes similarly to the WT. At the level of virulence, while we do not observe a statistical significance, our data clearly show a trend with a p value approaching <0.05 (Fig 5). Additionally, as described below we performed CAS agar (Fig 5E), HMV (fig. S5C), adhesion (fig S3D) and invasion (fig S3E) assays to show that hvKP2 and 94 aerobactin mutant behaves similarly to hvKP1 aerobactin mutant providing further strength to our studies. The high virulence of hvKP2 is brought up in the results (Ln 256-257) and in the discussion section (Ln 402-405).**

Third, results related to hvKP94 are particularly difficult to interpret, as Russo et al. previously published this strain as a partially virulent isolate that produced very little or no siderophores under iron-limiting conditions ([https://doi.org/10.1128/msphere.00045-21](https://urldefense.com/v3/__https:/doi.org/10.1128/msphere.00045-21__;!!Kv7QgGdTlhIaqSqT!MFuTqxE46KKc6cuFFDLxZvChgamOMZhHDGTG5pwXmVFg59HMotCBpZx03gFJ800zuGflZgxlFKxGDgr_8yLm6w07RA$)). Given this background, the expectation would be that an iucA- mutant would have no effect on pathogenesis since this strain is not producing meaningful amounts of siderophores to begin with. These flaws and overall lack of discussion of these discrepancies significantly undermine the authors' claims about the conservation of aerobactin-mediated effects across different hvKP genetic backgrounds. It remains clear that there is heterogeneity among hvKP isolates and the requirement of aerobactin for high levels of virulence is limited in scope. Conclusions could be strengthened by testing these strains and their corresponding iucA- mutants in some of the in vitro assays such as CAS, hmv, adhesion, invasion, and translocation.

**We thank the reviewer for suggesting assays to further strengthen our conclusions. Based on their suggestion we performed CAS agar assays (Fig 5E) that showed differences between the WT strains and their respective *iucA* mutants. HMV spin down assays under iron limited conditions further demonstrated that aerobactin mediated iron acquisition is important for the HMV phenotype for both hvKP2 and 94 (Fig. S5C). Third, we performed adhesion and invasion studies using Caco-2 cells and show that hvKP2 and 94 aerobactin mutants have a defect in our adhesion and invasion assays (Fig S3D-E). While there is strain to strain differences but overall the new assays performed are in line with our data with hvKP1 and its isogenic *iucA* mutant. Collectively, these new data further strengthen our results.**

• The authors' conclusions about translocation in the GI model rely heavily on an in vitro Caco-2 model that demonstrates a translocation defect at only a single timepoint (30 minutes) with highly variable results. While TEER measurements were extended to 6 hours post-infection, the translocation assay itself was limited to a single early timepoint. These conclusions would be significantly strengthened by:
o Including multiple timepoints for translocation assessment
o Testing the complemented strain to confirm the specificity of the observed defect
o Additionally, to strengthen their conclusion that aerobactin contributes to translocation across distinct hvKP lineages, the authors would benefit from performing additional transwell assays using the hvKP2 and hvKP94 strains and their respective mutants

**Based on the authors suggestion we do show multiple time points (up to two hours) with hvKP1 and *iucA*- mutant (Fig 6C), and the complemented strain (Fig S4A-D). Our plasmid complement strain restores the ability of the *iucA* mutant to translocate as efficiently as the WT strain, suggesting that the defect observed is corrected with the expression of aerobactin. This together with our adhesion and invasion assays with the complemented plasmid (Fig S3F-G) validate our cell culture results with hvKP1 and the isogenic *iucA* mutant.**

**As we believe that the translocation defect is due at the step of adhesion, we also performed adhesion and subsequently invasion studies with both the hvKP2 and 94 and their isogenic iucA mutants ( Fig S3D-E). These results are in accordance with our results with hvKP1 and the *iucA* mutant.**

• The authors suggested increased expression of rmpD may be causing the observed defect in adhesion and thus poor translocation of the iucA- strain. Because the rmpD- and ΔiucA rmpD- strains have high levels of adhesion, then including these strains in the GI model or transwell experiments would clarify if the phenotypes caused by the elevated expression of rmpD is in fact contributing to a translocation defect. i.e., rmpD mutant adheres better does this improve translocation?

**The role of *rmpD* in gut colonization and translocation is currently being separately considered. Furthermore, we would like to direct the reviewer to the study by *Walker et al.* (PMID32963003) where they showed using J774A.1 cells that compared to the WT strain the *rmpD* mutant had increased level of adhesion and invasion. We have added this in the result section in (Ln 335-336)**

**Part III – Minor Issues: Editorial and Data Presentation Modifications**
Please use this section for editorial suggestions as well as relatively minor modifications of existing data that would enhance clarity.

**Reviewer #1:**

- Figure S2 Bi should more clearly indicate which strains are spotted on the plate in which position.

**This has been corrected**

- Can the authors elongate the x-axis in Figure 1F? The data that is difficult to read due to variability and close proximity. The authors could also consider displaying data as a box plot or a violin plot to better visualize grouped data.

**We have elongated the x axis, but also provided higher resolution figures, which should help.**
- Figure 2. Can the authors speculate on why mice would have a lower survival rate in the presence of aerobactin but no differences in CFU?

**Figure 2 now figure 4, primarily focuses on the tail vein infection, where at 6 and 24 hours we do not observe any growth defects. However, as our *in vitro* growth assays suggest, under iron limiting conditions there is a growth defect that is attributed to *iucA*- mutant. We believe that over the course of infection in iron limited systemic sites compared to the WT the host has a better ability in controlling the mutants’ growth. Consequently, the WT through aerobactin has high virulence, whereas the mutant has reduced virulence and likely overtime is cleared by the host.**

**Reviewer #2:**

There is considerable redundancy in the iron acquisition systems of Kp, especially in vitro where high potency iron chelators are deployed to sequester any available Fe to test ‘essentiality’ for growth. Some attempts have been made to establish a hierarchy for these systems but their apparently redundant existence points to evolutionary drivers that are complex and which may sit outside of the human host. The broader Kp Species Complex (KpSC) is dominated by clinical isolates and the sequence database and strain collections are dominated by isolates from human disease. This may just be the ‘tip of the iceberg’ where this important family of bacteria are concerned.
Finding a growth defect in vitro for a iucA mutant, when the siderophore synthesis gene but not the rest of the aerobactin system allows mutant complementation in WT vs iucA- competition assays. This is currently a Supp figure but maybe the key panel could be added to Figure 1. This suggests that the quantity of aerobactin produced by the WT is sufficient to ‘feed’ both strains. It would have been interesting to see whether the aerobactin mutant has a phenotype in iron-free (not depleted) minimal medium, to which increasing concentrations of Fe are added. LB is a very rich medium and a poor test of the metabolic capacity of bacteria.

**The WT (hvKP1) and the isogenic mutant (iucA-) have been extensively characterized (PMID: 24664504; 26056379). Growth studies in M9 showed no difference, whereas in human specific media including urine and ascites fluid, which have low iron clearly demonstrated that aerobactin is required for optimal growth in those conditions.**

Hence, Line 158 should be “aerobactin synthesis is not required for colonisation”.

**Added synthesis**

Oral feeding leading to death (Fig. 1E) clearly demonstrates that the isolate is hypervirulent.

**hvKP1 has been demonstrated via other murine model of infections to be hypervirulent (PMID 24664504). We have modified the sentence to reflect the virulence.**

In the Fig. S2B(i) there seems to be a large difference in the siderophores produced by the iucA- mutant suggesting a lack of compensation by the remaining siderophores? This Figure could be better labelled. The transcription data in the same Supp figure is also telling – the apparent compensation by entC, iroB and irpB represented by transcript analysis (Fig. S2A), is not reflected in the CAS agar data (Fig. S2B).

**The figure has been relabeled to provide easier interpretation of the data.** **For context previous work by *Russo et al.* (PMID: 24664504) has demonstrated that in hvKP1 aerobactin is the primary siderophore produced (90%), and the other 3 make up the rest (10%). While there is some increase in *iroB* expression that likely does not compensate for the loss of aerobactin.**

In gut colonisation, is it possible that the apparent redundancy for iucA in colonisation is because only the iucA gene is deleted in this study, and that aerobactin is provided by the microbiome? Was the construction of a transporter mutant considered? The level of colonisation from a 10(6) dose without stomach acid neutralisation is another impressive demonstration of virulence. It would also be interesting to know whether the lack of ieuA impacts gut-mediated transmission in co-housing experiments; the phenotype missing from growth in the the gut lumen (Fig 1D), might be observed.

**As the receptor for aerobactin (*iutA*) is still present, it can likely uptake aerobactin or other iron bound siderophores as receptor promiscuity has been observed (PMID 39395422). We do not believe that aerobactin is being synthesized by the microbiota, as it is only present in *E. coli* and other Enterobacteriaceae. Our mice are specific pathogen free (SPF), and our recent microbiome study shows their gut microbiota does not contain other Enterobacteriaceae (PMID 39843522). Furthermore, our 1:1 coinfection growth study** (**Figure S1**), **show that aerobactin produced by the WT strain rescues the growth defect of the aerobactin synthesis mutant likely because of IutA.**

The observation regarding the lack of MLN colonisation (cf S. Typhimurium) is an interesting one, and likely correct. Salmonellae have an interesting relationship with phagocytes which may be absent in Kp, because the Kp capsule makes phagocytosis very challenging. However, there were some mice that had MLN colonisation, and others none - the effect looks binary.

**Thank you for pointing out the differences between the route of infections. We speculate that the mice that have some colonization in MLN, is likely either due to systemic infection, or the difficulty at times in harvesting MLN from the infected mice.**

Again relegating what are important and different mouse clearance kinetics of the WT and ieuA- strains to Supplementary data (S3) makes it unnecessarily tricky for the reader.

**We have now moved Figure S3 as a main Figure 2 to provide better accessibility.**

The data on the siderophore-negative strains are compelling though the interpretation that all are necessary (line 209) probably requires experimental support. It may be that aerobactin plus one other siderophore is sufficient.

**We agree with the reviewer that further studies are required to determine the hierarchy of the siderophores and other iron and metal uptake systems.** **The line 221 has been slightly modified.**

The deficiency in translocation in the ieuA isolate is well supported by the systemic infection data.
While the contribution from blood is likely to be limited (given the blood data, 2B; 2C), blood-rich organs like the liver and spleen should be perfused prior to analysis of bacterial counts. Was this done but not mentioned?

**We did not perform perfusion before determining bacterial count in specific organs. Thus, CFU from each organ likely includes some CFU from the blood. However, as we compare both the WT and the mutant colonization in organs homogenized similarly, we do not believe it impacts the overall analysis of our data.**

The data in Figure 2C suggest that aerobactin is not essential for bacterial growth in a sepsis model, as many more bacteria were recovered than injected, and this happened in the iucA mutant too. This is counterintuitive, though it may simply reflect siderophore redundancy. Was any thought given to trying to measure aerobactin levels in the blood of the mice, uninfected or infected to determine whether the microbiome is providing sufficient aerobactin to support growth of the bacterium that is unable to synthesize aerobactin?

**We do believe that other siderophores besides aerobactin likely contribute to the pathogenesis. We did not perform any assays to detect aerobactin in the bloodstream as that would require assay development through Mass Spectrometry to provide an exact measurement, which we believe is beyond the scope of this study.**

In Figure 3, subtle differences might be marked by too small an ‘n’. Which siderophore genes did the Kp strains in Figure 3 carry? Is there simple genomic evidence of strain diversity?

**These strains were chosen based on the differences in KL type, as KL type 1 and 2 are common hvKP isolates, with all of them containing an intact aerobactin locus. Additionally, hvKP94 was picked because of its partial virulence.** **Interestingly, hvKP2 (KL type 1) strain besides the 4 siderophores (enterobactin, Salmochelin, yersiniabactin and aerobactin), also contains the locus for colibactin (genotoxin). The presence of this other siderophore/toxin and possible capsule type might influence the increased virulence observed with this strain. Future studies will investigate the role of these factors in promoting increased virulence. We have added the siderophore produced by each strain in the strain table.**

Do we know that the key entry point/pathology for hvKp is the colon?

**Work by *Hsu et al.* (PMID: 25452552) demonstrated hvKP present in the lumen of the colon, and intracellular bacteria in the colonic epithelial layer of BALB/c mice. This work suggest that translocation possibly occurs while hvKP resides in the colon. Further studies are currently being undertaken to determine at the molecular level the likely site of translocation using our murine model of infection where highest colonization is observed in the cecum and the colon.**

Line 299 – wzi is thought to be involved in capsule retention, not synthesis

**We thank the reviewer for pointing this out. The sentence has been modified to reflect the role of Wzi.**

The study of encapsulation is made more difficult by the fact that strains with large capsules are hard to pellet and the pellet is very friable. Uronic acid after vigorous vortexing (assuming the capsule contains a uronic acid) is a direct way of measuring capsule production, were not different yet the assays for gene transcription suggested a difference. The effects of aerobactin on transcription of the genes assessed can be direct or indirect – direct is implied but the level of transcription of all the genes tested seems to be affected to a similar level (Fig 5B), or near similar level (Fig. 5C). This doesn’t spell specificity. It would be comforting that this wasn’t a general effect resulting from a higher level function (e.g. a low iron stress response) to see 2-3 housekeeping that were not impacted by the mutation. GyrA is mentioned but the Ct values or some more primary data measure should be presented, to facilitate reproduction and to see the actual levels of transcript being detected.

**We have included *rpoD* expression as a control (Fig 7B). Furthermore, all the Ct values are also now provided in the supplemental table.**

In Fig. 5F, it is hard to see how a 3 vs 3 comparison with considerable overlap could yield such a high P-value.

**For all cell culture studies we have now included all the replicates with statistics run on the pooled data. We have further provided data for each run as a supplemental table.**

Discussion

The interpretation is clear and reasonable, until attributing cause-effect (line 394 onwards). It is well established that capsule thickness is negatively correlated with adhesion and hence the reductions in invasion, which were consistent with the reduction in adhesion, might simply result from increased expression of the capsule. The transcription analysis would be better served by an RNASeq approach, rather than selected gene analysis. An unbiased analysis would show what specific functions are affected, or whether it is a more general transcriptional response to stress caused by reduced iron.

**We agree with the reviewer that unbiased approach is further needed to understand metal homeostasis at global level to understand the metabolic and other changes that occur due to metal stress. These are studies that are currently ongoing but beyond the scope of this work. To provide further context we have included housekeeping gene *rpoD* (Fig 7B) in our analysis and also provided other raw qRT-PCR data in supplemental table.**

The Figures are well presented but the data is typically highly processed in most cases and often referenced back to % of the WT. This does not readily allow a deeper understanding by the reader as **relative measure are subject to changes in either the numerator or denominator.**

**Raw data is now provided as a supplemental table in an accessible format, allowing for it to be analyzed in different ways.**

General

There are some key data to the arguments presented currently residing in Supplementary Data, and some interesting data too that, though not germane to the core argument, provide some insights into the aerobactin mutant. The reviewer appreciates that a line must be drawn but having to access Supplementary data to support key conclusions is a frustration. Given the online journal format, it should be possible to add in some of the positive data found in the Supps to the manuscript, without incurring much in costs.

**While we understand the frustration in reading the manuscript with supplemental figures in another document, we had to strike a fine balance in including what we deemed to be critical data, and made an editorial decision not based on cost but rather to have a tight focus on the key points of this manuscript. Based on suggestions, we have added new data to be included in the main manuscript and moved figure S3 into the main document as Fig 2 and also separated figures to provide easier reading.**

Fore example, iven the data in Fig S1A, the competition assay (Fig. S1B) is a clear demonstration that sufficient arobactin is produced to support full growth of iucA- bacteria.
The statistical analysis too has some anomalies – while the data in Fig. S5A mrkA is ‘ns’ without any significant overlap in the data, the Data for the complementation (Fig. S4.D) has significant overlap yet is strongly statistically different. Statically significant comparisons of 3 biological replicates with 3 biological replicates should not allow for any overlap of the three samples.

**The cell culture data now includes all the technical replicates from run to run, and statistics analysis is performed on this data.**

In the strains table, the siderophore intact genes should be listed, at least for the wild type. Is the parent strain generally available.

**In the strain table, we now list the siderophores that are expressed by the individual WT isolates. Additionally, the hvKP1 strain is available upon request.**

**Reviewer #3:**

 1. Lines 91-95 could be expanded to include greater detail as this section is critical information for this study.

**We have provided additional details about the loci that are present on the virulence plasmid, included how these virulence determinants allow hvKP to overcome complement and Lcn2. Ln 91-100)**

2. Line 157-159: To improve clarity for the reader, the authors could include a sentence stating that because fecal shedding reflects the number of bacteria present in the gut lumen, equivalent shedding between wild-type and mutant strains suggests that both are similarly capable of colonizing the gastrointestinal tract.

**We have added a sentence to provide further clarity.** (ln 164-166)

3. Figure 1H-I: Really nice data.

**We thank the reviewer for appreciating our results.**

4. Line 181-182: ‘We inoculated mice at a 1:1 ratio of the WT and the mutant strain, and as expected both strains colonized equally well in the GI tract.’ It looks like the WT outcompeted the mutant in the gastrointestinal samples (F: fecal, CE: cecum, CO: colon). Were there stats performed here?

**We thank the reviewer to bringing this to our attention. Statistical analysis was performed** **(Wilcoxon matched-pairs signed rank test). It was not listed in the figure legend which has now been corrected.** **We observed significance at some gut sites but not in fecal shedding, we bring this up now in the result section (Ln 192-197). This figure has also now been moved into the main document (Fig 2)**

5. Fig. S2Bi was difficult to see - more labeling needed to improve clarity. Also, more description in the figure legends would help improve clarity. Perhaps excising the halos and inserting them above the bar graph in Fig. S2Bii would help?

**Thank you for that suggestion. We excised the halo’s as suggested by the reviewer and added them on top of the bar graph to provide further clarity.**

6. I have no issues assessing the data, but the figures are low resolution even in the downloaded pdf.

**We have provided high resolution files separately, which should resolve the resolution issue.**

7. The data in SFig3i-ii is very important. The authors should consider moving it to a main figure.

**We agree with the reviewer and now have moved figure S3 into the main text.**

8. Fig4A-B: really nice data.

**We appreciate the positive comment on our results.**

9. Line 282-283: Additionally, T3F bind to abiotic catheters and facilitate UTIs in mice (Murphy et al. Role of Klebsiella pneumoniae type 1 and type 3 fimbriae in colonizing silicone tubes implanted into the bladders of mice as a model of catheter-associated urinary tract infections. Infect Immun. 2013;81(8):3009-17).

**We have added these references and modified the sentences (Ln 309).**

10. Line 289-290: ‘These results suggest that the reduced epithelial cell engagement observed in the iucA- mutant is likely independent of fimbrial gene expression.’ Is it possible that the fimbriae are involved in adherence but this is not evident at the transcriptional level? If fimbriae expression is activated in the WT and iucA mutant under iron deplete conditions, but HMV is only reduced in the WT, perhaps this helps expose the fimbriae to mediate adherence. The authors could consider discussing this or testing adherence of the fimbriae mutants under iron deplete conditions.

**We do believe that fimbriae contribute to hvKP interactions with host cells and other surfaces and thus would assume that deletion mutants would impact adhesion to host cells, which has been previously demonstrated (PMID 18474655; 40768400). We did not observe any changes transcript levels between the WT and *iucA*- mutant for the fimbriae genes but did observe differences with the CPS and HMV associated genes leading us to focus on those cell surface structures and observing corresponding changes in HMV, which impacts adhesion. However, we have modified the discussion Ln 440-444 to highlight the adhesive property of fimbriae.**

11. Fig5E: The data here is expressed as % adherence to WT. It would be nice to see the raw adherence data (even as supplemental) to get a sense of the % of bacteria that adhere to host cells under iron deplete conditions. It would be nice to compare this to the raw data under iron replete conditions.

**The raw data is now provided for all figures in an accessible format as a supplemental table.**

12. The discussion is accessible and enjoyable to read. But given how this study has highlighted the importance of aerobactin in mediating translocation from the gut, it would be useful to add a sentence to discuss if there may be a benefit to screening at risk patients for Kp strains that express aerobactin in their gut, particularly if they will be / have been administered antibiotics.

**We have added a sentence highlighting the potential of aerobactin as a molecular marker in a clinical setting. (Ln481-483)**

**Reviewer #4:**

• The introduction would benefit from a more balanced discussion of aerobactin's role in hvKP virulence. The current text presents aerobactin as consistently associated with enhanced virulence, but this oversimplifies the literature. I recommend the authors acknowledge several important discrepancies:
1. Convergent isolates: Some isolates that acquire aerobactin through horizontal gene transfer do not exhibit the expected hypervirulent phenotype, suggesting aerobactin alone is insufficient for hypervirulence.
2. Aerobactin-negative hvKP: Certain hvKP strains lacking aerobactin have been documented to cause severe invasive infections, indicating heterogeneity in iron-homestasis mechansims among hvKP.
3. Retained virulence in mutants: Previous reports have described aerobactin-deficient mutants that maintain significant virulence, contradicting the notion that aerobactin is "crucial" for pathogenesis.
**We agree with the reviewer that while aerobactin is important, strains lacking it, convergent strains or mutants, including our tail vein infection results, demonstrate partial virulence. We believe that the points raised by the reviewer are important but would be more appropriate in the discussion section, where we have included a paragraph detailing certain caveats to our study, including not all hvKP isolates encode for aerobactin, and convergent strains have been observed to have reduced virulence while they still encode for aerobactin, suggesting that for full virulence other factors might also be contributing. (Ln 461-474)**

Minor Comments:

• Ln 114-116: “..aerobactin is not necessary for gut colonization, it is crucial for translocation..” this should be softened, the iucA- mutant does get into extraintestinal organs in ~50% of mice.

**The language has been changed to “contributes to the translocation process...”**

• Ln 140- 143: “a more pronounced upregulation of the other siderophores..” This part of the sentence should be modified, only salmochelin displayed a statistically significant increase. Enterobactin and yersiniabactin expression were not increased in the iucA- mutant.

**The sentence has been modified to say: “Loss of aerobactin did not result in an increase in expression of other siderophores except for salmochelin (*iroB*) expression displaying a significant 4-fold increase, relative to the WT.”**

• Ln 149-151 and Fig 1C: “.. samples revealed elevated levels of all four siderophores..” Are iroB and irp2 statistically significant? It may also be worth noting that these expression levels are a fraction of those in iron-depleted media. Which could be related to the challenges of in vivo qRT-PCR or could suggest that the gut while iron limiting is not as limited as iron-depleted LB (which could explain why the iucA- mutant colonizes the GI tract as well as the wild type).

**We did not observe any significance in upregulation iroB and irp2 when comparing to the gyrase. As the reviewer suggested this could be due to difficulty in isolating bacterial RNA from the gut, but also could be due to the comparative analysis (in vivo versus in vitro). However, as aerobactin is the primary siderophore for hvKP1 (90% of all siderophores), having its expression high compared to the other siderophores is in line with that data.**

• Ln 179-185: “We speculate that, compared to the gut, the differences observed in systemic sites could be due to the infection barriers present within the host.” I agree, but I think this should be expanded upon in the discussion. For example, co-colonization data in the GI model (Fig S3A) suggests a tight bottle neck and founder effect in disseminated tissues.

**We appreciate the reviewer pointing out this interesting piece of data. We have expounded upon it further in the discussion section. (Ln 375-381)**

• Ln 277 “Aerobactin regulates the HMV phenotype in hvKP” Please be careful when describing the role of aerobactin, aerobactin does not directly “regulate” the HMV phenotype, it binds iron in the extracellular environment and brings it back to the cell. Expression of rmp is modulated by Fur. In a manuscript defining a new role for aerobactin it is important to be clear in technical writing so as not to be misinterpreted. Suggest something like “Aerobactin mutants have increased HMV”.

**We agree with the reviewer and have modified the heading.**

• Ln 394-415: As stated, these cell surface factors are known to be regulated by Fur. These changes in the aerobactin mutant seem to be global and the result of iron-deprivation. Since Fur repression is mediated by the availability of iron, some discussion / speculation should be included here about why expression of other siderophores is not sufficient to provide the cell with enough iron. Previous data from Russo et al., ([https://doi.org/10.1128/iai.01667-13](https://urldefense.com/v3/__https:/doi.org/10.1128/iai.01667-13__;!!Kv7QgGdTlhIaqSqT!MFuTqxE46KKc6cuFFDLxZvChgamOMZhHDGTG5pwXmVFg59HMotCBpZx03gFJ800zuGflZgxlFKxGDgr_8yLabR5ghQ$)) suggested that hvKP1 and its corresponding iucA- mutant do not produce any additional siderophores (by HPLC), despite their biosynthetic enzymes being expressed, as shown in this manuscript.

**Thank you for pointing out the *Russo et al.* study. The strain hvKP1 and the isogenic mutant *iucA::kan* used in the current study are those first described in that manuscript. In their data under various growth conditions, they demonstrated that while aerobactin is the primary siderophore (90%), other siderophores are also produced albeit at a lower level (10%). Some of these details have now been included in the discussion section. (Ln 434-438)**

• Ln 613 and 1009: The author’s state the transwell assay in Figure 4C shows the averages of 3 independent experiments. However, there are 4 datapoints per strain. Please clarify.

**More than 3 independent experiments were performed. We have corrected the figure legend to reflect it.**

• Figure images are blurry.

**High resolution Images are provided with the revised manuscript.**

• Please provide raw data for all experiments as a supplemental table: Inoculum for all mouse infections, inoculum and CFU values for top and bottom layers for translocation assays, raw data for normalized figures such as Fig 4A-B and 5D-F, etc.

**Raw data has been provided now as a supplemental table.**

• Please update the methods for animal experiments to discuss randomization and blinding of **experiments to comply with ARRIVE guidelines, a requirement of PLOS Pathogens.**

**These details have been added to the method section**

• If mice were euthanized due to a loss of >=20% initial body weight, please provide body weight measurements as a supplemental table.

**Body weight information is now included in the supplemental table.**

PLOS authors have the option to publish the peer review history of their article ([what does this mean?](https://urldefense.com/v3/__https:/track.editorialmanager.com/CL0/https:*2F*2Fjournals.plos.org*2Fplospathogens*2Fs*2Feditorial-and-peer-review-process*23loc-peer-review-history/1/010f0199008d1887-97224a87-6071-4e5d-9e12-62848924216b-000000/MhRYvgujzwAapuC-cd5ZAe-8uG3tfMMPn46CZpiKxT0=226__;JSUlJSUl!!Kv7QgGdTlhIaqSqT!MFuTqxE46KKc6cuFFDLxZvChgamOMZhHDGTG5pwXmVFg59HMotCBpZx03gFJ800zuGflZgxlFKxGDgr_8yJ4QGZQlw$)). If published, this will include your full peer review and any attached files.


If you choose “no”, your identity will remain anonymous but your review may still be made public.


**Do you want your identity to be public for this peer review?** For information about this choice, including consent withdrawal, please see our [Privacy Policy](https://urldefense.com/v3/__https:/track.editorialmanager.com/CL0/https:*2F*2Fwww.plos.org*2Fprivacy-policy/1/010f0199008d1887-97224a87-6071-4e5d-9e12-62848924216b-000000/JNNQ9_qemJvuvdGUHNOramY_iG4Ek0qY63LcMD_z43c=226__;JSUl!!Kv7QgGdTlhIaqSqT!MFuTqxE46KKc6cuFFDLxZvChgamOMZhHDGTG5pwXmVFg59HMotCBpZx03gFJ800zuGflZgxlFKxGDgr_8yLPEIFEdQ$).

Reviewer #1: No

Reviewer #2: **Yes:** Richard Strugnell

Reviewer #3: No

Reviewer #4: No

[NOTE: If reviewer comments were submitted as an attachment file, they will be attached to this email and accessible via the submission site. Please log into your account, locate the manuscript record, and check for the action link "View Attachments". If this link does not appear, there are no attachment files.]

**Figure resubmission:**

While revising your submission, we strongly recommend that you use PLOS’s NAAS tool ([https://ngplosjournals.pagemajik.ai/artanalysis](https://urldefense.com/v3/__https:/ngplosjournals.pagemajik.ai/artanalysis__;!!Kv7QgGdTlhIaqSqT!MFuTqxE46KKc6cuFFDLxZvChgamOMZhHDGTG5pwXmVFg59HMotCBpZx03gFJ800zuGflZgxlFKxGDgr_8yJ4xVCJuQ$)) to test your figure files. NAAS can convert your figure files to the TIFF file type and meet basic requirements (such as print size, resolution), or provide you with a report on issues that do not meet our requirements and that NAAS cannot fix.

After uploading your figures to PLOS’s NAAS tool - [https://ngplosjournals.pagemajik.ai/artanalysis](https://urldefense.com/v3/__https:/ngplosjournals.pagemajik.ai/artanalysis__;!!Kv7QgGdTlhIaqSqT!MFuTqxE46KKc6cuFFDLxZvChgamOMZhHDGTG5pwXmVFg59HMotCBpZx03gFJ800zuGflZgxlFKxGDgr_8yJ4xVCJuQ$), NAAS will process the files provided and display the results in the "Uploaded Files" section of the page as the processing is complete. If the uploaded figures meet our requirements (or NAAS is able to fix the files to meet our requirements), the figure will be marked as "fixed" above. If NAAS is unable to fix the files, a red "failed" label will appear above. When NAAS has confirmed that the figure files meet our requirements, please download the file via the download option, and include these NAAS processed figure files when submitting your revised manuscript.

**Reproducibility:**

To enhance the reproducibility of your results, we recommend that authors of applicable studies deposit laboratory protocols in protocols.io, where a protocol can be assigned its own identifier (DOI) such that it can be cited independently in the future. Additionally, PLOS ONE offers an option to publish peer-reviewed clinical study protocols. Read more information on sharing protocols at [https://plos.org/protocols?utm_medium=editorial-email&utm_source=authorletters&utm_campaign=protocols](https://urldefense.com/v3/__https:/plos.org/protocols?utm_medium=editorial-email&utm_source=authorletters&utm_campaign=protocols__;!!Kv7QgGdTlhIaqSqT!MFuTqxE46KKc6cuFFDLxZvChgamOMZhHDGTG5pwXmVFg59HMotCBpZx03gFJ800zuGflZgxlFKxGDgr_8yJ6DGUkeQ$)
