# Supplementary material for: Aerobactin is a key driver of hypervirulent Klebsiella pneumoniae translocation and virulence
Source: PLoS Pathog. 2026 Apr 13;22(4):e1014122. doi: 10.1371/journal.ppat.1014122 (PMC13089870; doi:10.1371/journal.ppat.1014122)
Supplement: Attachment — Submitted filename: Author rebuttal-3.24.26.docx [file ppat.1014122.s012.docx]

PPATHOGENS-D-25-01441R1
Aerobactin is a key driver of hypervirulent *Klebsiella pneumoniae* translocation and virulence
PLOS Pathogens

Dear Dr. Zafar,

Thank you for submitting your revised manuscript to PLOS Pathogens. After careful consideration, we feel that while you have addressed many of the concerns from the reviewers, two reviewers feel that there are some still some minor issues to address, and I agree with them. We invite you to submit a revised version of the manuscript that addresses the points raised.

Please submit your revised manuscript by May 16 2026 11:59PM. If you will need more time than this to complete your revisions, please reply to this message or contact the journal office at [plospathogens@plos.org](mailto:plospathogens@plos.org). When you're ready to submit your revision, log on to [https://www.editorialmanager.com/ppathogens/](https://urldefense.com/v3/__https:/www.editorialmanager.com/ppathogens/__;!!I6g4XUvE7pGbSZtt6A!fPnOFahqUz_bM1Cocv7TWFd5BSL-tobrlnhFiKcmgdRZ6f8Z5gzbk4yJWOsLoKi7ZuObNfu-XgHYjqe8e-3HdFXHcg$) and select the 'Submissions Needing Revision' folder to locate your manuscript file.

Please include the following items when submitting your revised manuscript:

* A letter that responds to each point raised by the editor and reviewer(s). You should upload this letter as a separate file labeled 'Response to Reviewers'. This file does not need to include responses to any formatting updates and technical items listed in the 'Journal Requirements' section below.
* A marked-up copy of your manuscript that highlights changes made to the original version. You should upload this as a separate file labeled 'Revised Manuscript with Track Changes'.
* An unmarked version of your revised paper without tracked changes. You should upload this as a separate file labeled 'Manuscript'.

If you would like to make changes to your financial disclosure, competing interests statement, or data availability statement, please make these updates within the submission form at the time of resubmission. Guidelines for resubmitting your figure files are available below the reviewer comments at the end of this letter.

We look forward to receiving your revised manuscript.

Kind regards,

Leigh Knodler
Academic Editor
PLOS Pathogens

Thomas Guillard
Section Editor
PLOS Pathogens

Sumita Bhaduri-McIntosh
Editor-in-Chief
PLOS Pathogens
orcid.org/0000-0003-2946-9497

Michael Malim
Editor-in-Chief
PLOS Pathogens
orcid.org/0000-0002-7699-2064

**Journal Requirements:**

If the reviewer comments include a recommendation to cite specific previously published works, please review and evaluate these publications to determine whether they are relevant and should be cited. There is no requirement to cite these works unless the editor has indicated otherwise.

1) Please ensure that the CRediT author contributions listed for every co-author are completed accurately and in full.
At this stage, the following Authors/Authors require contributions: Md. Maidul Islam, and Suhrid Maiti. Please ensure that the full contributions of each author are acknowledged in the "Add/Edit/Remove Authors" section of our submission form.
The list of CRediT author contributions may be found here: [https://journals.plos.org/plospathogens/s/authorship#loc-author-contributions](https://urldefense.com/v3/__https:/journals.plos.org/plospathogens/s/authorship*loc-author-contributions__;Iw!!I6g4XUvE7pGbSZtt6A!fPnOFahqUz_bM1Cocv7TWFd5BSL-tobrlnhFiKcmgdRZ6f8Z5gzbk4yJWOsLoKi7ZuObNfu-XgHYjqe8e-2-9QSuZg$)

2) We do not publish any copyright or trademark symbols that usually accompany proprietary names, eg ©,  ®, or TM  (e.g. next to drug or reagent names). Therefore please remove all instances of trademark/copyright symbols throughout the text, including:
- TM on page: 25.

3) Thank you for including an Ethics Statement for your study. Please include:
i) A statement that formal consent was obtained (must state whether verbal/written) OR the reason consent was not obtained (e.g. anonymity). NOTE: If child participants, the statement must declare that formal consent was obtained from the parent/guardian.].

4) Please amend your detailed Financial Disclosure statement. This is published with the article. It must therefore be completed in full sentences and contain the exact wording you wish to be published.
1) State what role the funders took in the study. If the funders had no role in your study, please state: "The funders had no role in study design, data collection and analysis, decision to publish, or preparation of the manuscript."
2) If any authors received a salary from any of your funders, please state which authors and which funders..
If you did not receive any funding for this study, please simply state: u201cThe authors received no specific funding for this work.u201d

5)  Please ensure that the funders and grant numbers match between the Financial Disclosure field and the Funding Information tab in your submission form. Note that the funders must be provided in the same order in both places as well.

**Reviewers' Comments:**

Reviewer's Responses to Questions

**Part I - Summary**
Please use this section to discuss strengths/weaknesses of study, novelty/significance, general execution and scholarship.

Reviewer #2: The authors have addressed many of the key issues. The attempts at complementation were unsuccessful with the gene (operon structure< regulation?) but there was some short term complementation in vivo using a plasmid carrying multiple genes. Complementation is tricky but the purpose is to lend weight to conclusions. If the authors are unable to complement the bacteria with construct that is stable in vivo, it is very hard to be dogmatic that aerobactin is the key product missing from the mutants.

The data presented in the rebuttal Figure are supportive but the loss of plasmid in vivo is not well explained. If it is simply a replicon instability issue then there are multiple replicons with strict (vs relaxed) replication controls that should be retained.

**We acknowledge the reviewer’s concerns and would like to provide further information that we hope will provide clarification to observed phenotypes. The Plasmid pFUS2 used in the studies is based on the pQE-30 vector, which carries the ColE1 origin of replication, a relaxed control mechanism that allows for a reasonably high copy number. This plasmid was chosen because it has been demonstrated to be compatible with *K. pneumoniae,* particularly the hvKP1 isolate used in the study, and confers gentamicin resistance (PMID 24664504). We were able to successfully demonstrate that this plasmid allows for complementation with multiple assays, including CAS agar (fig S3H), HMV (Fig. S5D), adhesion (Fig S3F), invasion (Fig S3G), and translocation (Fig S4 A-D), where the plasmid complemented the defect of the *iucA*- strain in the presence of gentamicin. Under *in vivo* conditions, when the mice were orally inoculated with hvKP *iucA*- containing the pFUS2(*iucA-D*), it was poorly retained and lost within 24 hours post-inoculation. These results suggest that without the presence of gentamicin to retain the plasmid under *in vivo* conditions, the plasmid is lost rather quickly (this data was included with the previous rebuttal document).**

**To bolster our *in vivo* results, we tested two other distinct isolates (hvKP2 and hvKP94) and their respective aerobactin mutants, which exhibited similar phenotypes to hvKP1 and its isogenic *iucA* mutant both *in vivo* and *in vitro*. We believe these results strongly suggest that the phenotypes observed are due to aerobactin. Lastly, we have added a caveat section in the discussion, outlining the limitations of our studies, which includes difficulty in *in vivo* complementation (Line 463-478). Taken together, we hope that this would alleviate the reviewer concerns.**

Reviewer #4: This revised manuscript prepared by Hernadez et al., is very much improved. Specifically with regards to scientific rigor and robust support of their conclusions. However, some issues remain requiring clarification of humane endpoints utilized in mouse studies, and some slight misinterpretation of the published literature which could modify the tone and narrative of the discussion.

**Part II – Major Issues: Key Experiments Required for Acceptance**
Please use this section to detail the key new experiments or modifications of existing experiments that should be absolutely required to validate study conclusions.

Generally, there should be no more than 3 such required experiments or major modifications for a "Major Revision" recommendation. If more than 3 experiments are necessary to validate the study conclusions, then you are encouraged to recommend "Reject".

Reviewer #2: The issue around the large MOI is not well addressed – if the same MOI is used in other studies then both might be criticized. The purpose of the translocation study was to show that the bacteria can move through a cell monolayer. Ignoring the impact of centrifugation, if in vivo there are good examples where there are 50 bacteria for every cell in the epithelial barrier then such an MOI might be justified. Otherwise the studies risks observation of an artefact resulting from using very high bacterial numbers.

Out of interest, were lower numbers of bacteria ever used? Lacking classical bacterial epithelial ‘invasion’ processes like Type 3 secretion systems, Kpn will need some other mechanism for creating changes to the cell membrane seen is either invasion or paracellular egress. The suggestion of using microscopy was to ask the question whether Kpn are seen within, or between, cells.

**We thank the reviewer for this thoughtful critique regarding the MOI and the potential for artifacts. We agree that visual confirmation via microscopy is a valuable approach for observing these interactions. To address the concern that the high number of bacteria might be creating an artifact, we have relied on our TEER analysis (Figure 6D). The stability of the epithelial barrier during the 30-minute and 2-hour timepoints in which translocation was being measured indicates that the bacteria were interfacing with an intact epithelium rather than via an artifact of non-specific damage. Regarding the use of fluorescence to visualize internalization, studies such as Hsu *et al.* (PMID 25452552) have already established via confocal microscopy and fluorescence that *K. pneumoniae* adheres to and internalizes into epithelial cells using Caco-2 HCT-8 and HT-29 at similar MOIs used in our studies. We believe our combination of TEER stability and the 1000-fold difference between adhesion (~1%) and invasion (~0.004%) provides strong evidence that the epithelial barrier remains a functional hurdle at this MOI. Furthermore, our results demonstrate that epithelial barrier function is not affected under *in vitro* and *in vivo* conditions, and that hvKP1 translocates through the transcellular route, results which are in line with the *Hsu et al*. study. Nonetheless, based on reviewer’s concern, we have added *in vivo* visualization of hvKP invading epithelial layer as a caveat to our studies and have added that to the discussion section as it would further provide further evidence and credence to our *in vitro* cell culture assays (Line 476-478).**

**In reference to the reviewer’s interest in lower bacterial numbers, we did not test lower MOIs in the current study. This decision was based on the low efficiency of the invasion process, which presented a methodological necessity for an MOI of 50. While the adhesion assay results in about 1% of the inoculum recovered, the invasion assay only yields about 0.004% of the inoculum. Reducing the MOI further would potentially push these invasion counts below the limit of detection, introducing potential sampling error and making it statistically challenging to distinguish biological differences between the WT and mutant strains (Figure 6A-B). If the high bacterial load were causing an artifactual breakdown of the epithelial cells, we would expect to see a much higher percentage of the adhered cells invading or translocating.**

**Furthermore, an MOI of 50 is consistent with established *K. pneumoniae* literature, where ranges of 30-100 are standard (PMID 11083790; 25452552; 40028183; 27534887; 41753764; 41304276), and some studies utilize MOIs as high as 200-1000 (41828398; 19650888). Finally, we agree that the mechanism of interaction in the absence of a T3SS is a compelling area of study. We showed in this manuscript that fimbriae expression was not altered by the loss of aerobactin (Figure S5) and are currently investigating whether the type 6 secretion system effector proteins may mediate the invasion process.**

Reviewer #4: 1. Translocation defect / serum survival: While it is true that complement is likely the first line of defense Klebsiella pneumoniae faces post translocation, the serum killing assay as performed does not support or refute the conclusion that “the decreased number of translocated bacteria in mutant are not due to host clearance mechanism at an early timepoint, but rather a defect in translocation.” While I have concerns about the serum killing assay data as presented and the experimental methods, the in vitro translocation data presented in Fig 6C and S4A-B are convincing and demonstrate a clear translocation defect in the hvKP1 iucA mutant that has been restored by expressing iucABCD on a plasmid. Discussion around the results of the serum killing assay should be tempered as a siderophore mutant is unlikely to be more sensitive to 10% rabbit serum than WT. Serum killing assays are usually done with higher concentrations of serum. For example, a previous study found that using hvKP1 ΔiucA there was a growth / survival defect in 90% human serum compared to WT (PMID 26056379). Regardless, conclusions surrounding the translocation defect are supported directly by other experiments.

**We acknowledge the reviewer's concern regarding differences in complement concentrations and the source of complement. It is suggested that rabbit serum is generally more potent, especially as it contains higher levels of complement proteins, which is why 10% rabbit serum was used in our assay (PMID 6403466; 6995545; 8565784). The composition of our complement-killing assay also differs from that of the other study. We recently established that the KPPR1S capsule-deficient strain is sensitive to 10% rabbit serum and not heat-inactivated serum, providing us with the rationale to use the specific assay (PMID 41670378). We incubated our strains for up to 1 hour, and as the reviewer rightly points out, it is possible that a longer incubation time might reveal a growth defect in serum for the aerobactin mutant, as was observed at 24 hours in the other study (PMID 26056379). Therefore, based on the reviewer’s suggestion, we have tempered our language in the results section (Line 242-244).**

2. Clarification of Humane Endpoint Criteria: The Methods state that mice were euthanized if they were unable to eat or drink, lost ≥20% of body weight, and exhibited respiratory distress. Are all three criteria necessary to justify euthanasia? The survival data in Figure 4 and corresponding weight loss data in TableS1_data show that mice were allowed to survive after losing ≥20% of body weight and in some cases for several days before being marked as a death. Because the survival curves in Figure 4 could differ substantially depending on how endpoints were applied, please clarify how humane endpoint criteria were utilized for these studies. If weight loss ≥20% of initial body weight is not a hard fast rule for euthanasia, as is typical for IACUC approved protocols, please clarify. With some strains I have observed mice losing significant weight while showing no other signs of infection. I believe that clarification on endpoint criteria should be sufficient to alleviate this concern. In future studies, utilization of a clinical scoring system by an individual blinded to the study and / or ensuring investigators are blinded to strain identity would further enhance transparency and eliminate ambiguity.

**We thank the reviewer for the opportunity to clarify our humane endpoint criteria. Per our approved IACUC protocol, euthanasia was triggered by the observation of clinical signs of moribundity. While our protocol lists individual signs, including weight loss, altered gait, hunched posture, and respiratory distress, these were applied as indicators of an irreversible clinical decline. As the reviewer noted, mice in these infection models can experience significant weight loss while remaining clinically stable. Our data (Supplemental raw data) shows instances where mice hit a weight loss threshold but remained active and subsequently recovered. In these cases, because the weight loss was transient and not accompanied by other clinical signs of distress, the animals did not meet the ‘moribund’ criteria for euthanasia and were instead closely monitored with increased frequency for signs of clinical distress. We have updated the Materials and Methods section (Lines 598-604) to clarify that these clinical observations guided our euthanasia decisions. We also appreciate the suggestion regarding blinded clinical scoring and will incorporate these practices into our future study designs.**


3. Discrepancies Between Survival and Weight Data: In the iucA group, Figure 4D indicates that four mice were alive at Day 8. However, the longitudinal weight table shows only three mice with recorded body weights at Day 8 (192 h). Please clarify whether one animal’s Day 8 weight measurement is missing from the table or whether there is a discrepancy between the survival and weight datasets. In addition, in the data for Figure 4D, deaths and weight loss measurements in the iucA mutant groups do not match. For example, mouse one was listed as dead on day 7 but there is weight loss data on day 8. Please clarify / resolve the discrepancies between the deaths in Figure 4D and the weight loss data.

**We thank the reviewer for identifying these discrepancies. Upon thorough review of the raw data, we have addressed these oversights as follows:**

- **Missing weight data: We confirm that mouse #7 was missing weight entries for days 6-8 in the original table. These values have now been correctly restored.**
- **Dataset alignment: We identified that mice #1 and #2 were inadvertently swapped between the survival curve (Figure 4D) and the weight loss dataset. We have corrected the weight loss measurements to ensure they accurately reflect the animal order presented in the survival curve.**
- **Consistency and formatting: To improve clarity, mice that reached the humane endpoint prior to the scheduled weigh-in are now consistently denoted as “ND” for that final time point indicating that the mouse was found moribund and not weighed. Furthermore, we have standardized all percent body weight values to one decimal place to ensure consistency across the dataset, as the original entries varied between 3-4 decimal places.**

4. Interpretation of Prior Siderophore Data (Russo et al.): The revised manuscript and accompanying responses state that, based on Russo et al. (PMID: 24664504), “aerobactin is the primary siderophore produced (90%), and the other 3 make up the rest (10%).”
This interpretation does not accurately reflect the data presented in the cited study. In Russo et al., 100% of detectable siderophore activity (by HPLC fractionation and CAS assay) was found within a single coarse HPLC fraction (fraction 5). Upon further sub-fractionation, approximately 90% of siderophore activity was localized to fraction 5c and ~10% to fraction 5d. Fraction 5c was chemically identified as aerobactin. However, fraction 5d was not structurally characterized as a distinct siderophore. The minor activity detected in fraction 5d more likely reflects an aerobactin-related species rather than a distinct siderophore system, as it co-fractionated with aerobactin and was not independently characterized. Importantly, the authors explicitly state: “As 5c and 5d elute in neighboring fractions, the activity in fraction 5d probably represents carryover from fraction 5c.”
Furthermore, in the ΔiucA strain, siderophore activity was no longer detected in fraction 5, and only minimal background activity was observed in fraction 1. Given that fraction 1 corresponds to early-eluting, highly polar, low–molecular-weight species under the reverse-phase conditions used, this weak signal is unlikely to represent production of a structurally defined siderophore such as enterobactin, salmochelin, or yersiniabactin. Rather, these data further support the conclusion that aerobactin is the sole functionally significant siderophore produced by hvKP1 under the tested conditions.

It is biologically plausible that a strain the produces high-levels of aerobactin may have its ability to produce the other siderophores diminished, even when their biosynthetic enzymes are present and expressed). Notably, yersiniabactin and enterobactin (and thus salmochelin) share the same chorismate precursor while aerobactin biosynthesis utilizes a citrate backbone.

While it is possible that the HPLC conditions utilized in the referenced study were not optimal for resolving all potential siderophores, and thus they were missed. The results of the referenced study do not suggest that “aerobactin is the primary siderophore produced (90%), and the other 3 make up the rest (10%).” Rather the data show that all detectable siderophore activity in hvKP1 co-fractionated with aerobactin under the tested conditions.

The in vivo phenotypes described in hvKP1 ΔiucA may therefore reflect deletion of the only functionally significant siderophore produced by this strain. In contrast, strains such as hvKP2, NTUH-K2044, and KPPR1 retain high virulence despite deletion or absence of iucA, suggesting strain-specific differences in functional redundancy of iron acquisition systems. This possibility should be discussed more explicitly.

**We appreciate the reviewer's detailed response regarding the study by *Russo et al*. (PMID: 24664504). As noted, the HPLC analysis suggests that aerobactin is the predominant siderophore produced by hvKP1. Nonetheless, they also conducted a quantitative siderophore production assay with hvKP1 and other hypervirulent isolates, and their corresponding *iucA* mutants, grown under two different conditions. Deletion of *iucA* in that assay results in a significant reduction in siderophore production, but not complete ablation. The 90/10 percent figure is based on the authors' suggestion. However, as the reviewer pointed out, this could be due to growth conditions. For this reason, we have added a qualifying statement noting that aerobactin is the primary siderophore (Line 107-110; 372-373).**

**Part III – Minor Issues: Editorial and Data Presentation Modifications**
Please use this section for editorial suggestions as well as relatively minor modifications of existing data that would enhance clarity.

Reviewer #2: (No Response)

Reviewer #4: The data related to diverse strains of hypervirulent Klebsiella (hvKP1, hvKP2, and hvKP94) strengthen their results and conclusions. hvKP94 is described as K1-ST23 in this study and in the prior publication from which the strain was obtained. Could the authors clarify how sequence type (ST) and capsule type (K/KL) were determined for hvKP94? Specifically, was whole-genome sequencing used for assignment, or were these designations based on prior serologic or PCR-based typing?

**We thank the reviewer for this question. The hvKP94 strain was characterized by the Russo Lab (University at Buffalo) using whole-genome sequencing (WGS) for its original description as a K1/ST23 lineage strain. While the source laboratory frequently employs PCR-based detection of hypervirulence-associated biomarkers, including certain capsule types, *iucA*, *iroB*, *peg-344*, *rmpA* and *rmpA2* (PMID 29925642; PMID 33762316), the ST23 and K1 designations were assigned based on their genomic characterization of this reference strain. We have updated the Materials and Methods section to reflect that these designations were derived from the original genomic analysis by the source laboratory (Lines 534-536). For consistency, we also updated the K2 designation of hvKP1 to KL2 and moved it to after the ST86 designation, in the Material and Methods section (Line 504).**

Line 401-403: “possibly due to expression of colibactin..” To me this seems unlikely, loss of iucA would be expected to impair iron scavenging under-host restricted conditions. Colibactin is a genotoxin and would not be expected to compensate for siderophore-mediated iron uptake. In addition, a previous study found that in NTUH-K2044 (which does not contain colibactin) an iucA deletion had no discernable effect on virulence albeit in a different animal model (PMID: 18433330). Is it more likely that while iuc is the primary siderophore produced in nearly all hvKP isolates, hvKP2 is better equipped to utilize its secondary siderophore systems than hvKP1?

**Thank you for raising the point regarding colibactin. We believe there is some confusion regarding our statement mentioning colibactin in the discussion section. The rationale for putting the statement is as follows: We raised colibactin speculatively, as CibA from the colibactin synthesis locus is involved in both enterobactin and yersiniabactin synthesis in pathogenic *E. coli* isolates (PMID 23853582). Additionally, we speculate that virulence associated with colibactin could overcome the lack of aerobactin (reduced virulence) in hvKP2. However, as the reviewer mentioned, it is entirely possible that the expression of siderophores in the hvKP2 and other isolates may differ, allowing them to compensate for the lack of aerobactin. We have modified the sentence to reflect the reviewer's point (Line 402-404).**

PLOS authors have the option to publish the peer review history of their article ([what does this mean?](https://urldefense.com/v3/__https:/track.editorialmanager.com/CL0/https:*2F*2Fjournals.plos.org*2Fplospathogens*2Fs*2Feditorial-and-peer-review-process*23loc-peer-review-history/1/010f019cfba6aef7-23e85f75-8a59-4869-9dbf-4b7ba4f4a73c-000000/KDw7koxzKBfFjPdy1jFmc6ScLJdv00NVC_f1_Co_ap4=254__;JSUlJSUl!!I6g4XUvE7pGbSZtt6A!fPnOFahqUz_bM1Cocv7TWFd5BSL-tobrlnhFiKcmgdRZ6f8Z5gzbk4yJWOsLoKi7ZuObNfu-XgHYjqe8e-0lZFAXxg$)). If published, this will include your full peer review and any attached files.


If you choose “no”, your identity will remain anonymous but your review may still be made public.


**Do you want your identity to be public for this peer review?** For information about this choice, including consent withdrawal, please see our [Privacy Policy](https://urldefense.com/v3/__https:/track.editorialmanager.com/CL0/https:*2F*2Fwww.plos.org*2Fprivacy-policy/1/010f019cfba6aef7-23e85f75-8a59-4869-9dbf-4b7ba4f4a73c-000000/a8Mm3RriYMBBfP4WTUJD5xbXyw8EV5ceb7cgQE9BvxE=254__;JSUl!!I6g4XUvE7pGbSZtt6A!fPnOFahqUz_bM1Cocv7TWFd5BSL-tobrlnhFiKcmgdRZ6f8Z5gzbk4yJWOsLoKi7ZuObNfu-XgHYjqe8e-2xR8f6dw$).

Reviewer #2: **Yes:** Richard Strugnell

Reviewer #4: No

**Figure resubmission:**

While revising your submission, we strongly recommend that you use PLOS’s NAAS tool ([https://ngplosjournals.pagemajik.ai/artanalysis](https://urldefense.com/v3/__https:/ngplosjournals.pagemajik.ai/artanalysis__;!!I6g4XUvE7pGbSZtt6A!fPnOFahqUz_bM1Cocv7TWFd5BSL-tobrlnhFiKcmgdRZ6f8Z5gzbk4yJWOsLoKi7ZuObNfu-XgHYjqe8e-2one8d4A$)) to test your figure files. NAAS can convert your figure files to the TIFF file type and meet basic requirements (such as print size, resolution), or provide you with a report on issues that do not meet our requirements and that NAAS cannot fix.

After uploading your figures to PLOS’s NAAS tool - [https://ngplosjournals.pagemajik.ai/artanalysis](https://urldefense.com/v3/__https:/ngplosjournals.pagemajik.ai/artanalysis__;!!I6g4XUvE7pGbSZtt6A!fPnOFahqUz_bM1Cocv7TWFd5BSL-tobrlnhFiKcmgdRZ6f8Z5gzbk4yJWOsLoKi7ZuObNfu-XgHYjqe8e-2one8d4A$), NAAS will process the files provided and display the results in the "Uploaded Files" section of the page as the processing is complete. If the uploaded figures meet our requirements (or NAAS is able to fix the files to meet our requirements), the figure will be marked as "fixed" above. If NAAS is unable to fix the files, a red "failed" label will appear above. When NAAS has confirmed that the figure files meet our requirements, please download the file via the download option, and include these NAAS processed figure files when submitting your revised manuscript.

**Reproducibility:**

To enhance the reproducibility of your results, we recommend that authors of applicable studies deposit laboratory protocols in protocols.io, where a protocol can be assigned its own identifier (DOI) such that it can be cited independently in the future. Additionally, PLOS ONE offers an option to publish peer-reviewed clinical study protocols. Read more information on sharing protocols at [https://plos.org/protocols?utm_medium=editorial-email&utm_source=authorletters&utm_campaign=protocols](https://urldefense.com/v3/__https:/plos.org/protocols?utm_medium=editorial-email&utm_source=authorletters&utm_campaign=protocols__;!!I6g4XUvE7pGbSZtt6A!fPnOFahqUz_bM1Cocv7TWFd5BSL-tobrlnhFiKcmgdRZ6f8Z5gzbk4yJWOsLoKi7ZuObNfu-XgHYjqe8e-1biogIJA$)

*In compliance with data protection regulations, you may request that we remove your personal registration details at any time.* [*(Remove my information/details)*](https://urldefense.com/v3/__https:/track.editorialmanager.com/CL0/https:*2F*2Fwww.editorialmanager.com*2Fppathogens*2Flogin.asp*3Fa=r/1/010f019cfba6aef7-23e85f75-8a59-4869-9dbf-4b7ba4f4a73c-000000/srcjlfoVp7P6GWrtscyC3LlcJKss3jJ4rvVgKHbysT4=254__;JSUlJSU!!I6g4XUvE7pGbSZtt6A!fPnOFahqUz_bM1Cocv7TWFd5BSL-tobrlnhFiKcmgdRZ6f8Z5gzbk4yJWOsLoKi7ZuObNfu-XgHYjqe8e-0MnEEcAg$)*. Please contact the publication office if you have any questions.*
